# Insulin‐like growth factor 1 deficiency exacerbates hypertension‐induced cerebral microhemorrhages in mice, mimicking the aging phenotype

**DOI:** 10.1111/acel.12583

**Published:** 2017-03-14

**Authors:** Stefano Tarantini, Noa M. Valcarcel‐Ares, Andriy Yabluchanskiy, Zsolt Springo, Gabor A. Fulop, Nicole Ashpole, Tripti Gautam, Cory B. Giles, Jonathan D. Wren, William E. Sonntag, Anna Csiszar, Zoltan Ungvari

**Affiliations:** ^1^ Reynolds Oklahoma Center on AgingTranslational Geroscience LaboratoryDepartment of Geriatric MedicineUniversity of Oklahoma Health Sciences CenterOklahoma CityOKUSA; ^2^ Department of PhysiologyUniversity of Oklahoma Health Sciences CenterOklahoma CityOKUSA; ^3^ Arthritis & Clinical Immunology Research ProgramOklahoma Medical Research FoundationOklahoma CityOKUSA; ^4^ Department of Biochemistry and Molecular BiologyUniversity of Oklahoma Health Science CenterOklahoma CityOKUSA; ^5^ Department of Medical Physics and InformaticsUniversity of SzegedSzegedHungary

**Keywords:** arteriole, dementia, gait dysfunction, microbleed, oxidative stress

## Abstract

Clinical and experimental studies show that aging exacerbates hypertension‐induced cerebral microhemorrhages (CMHs), which progressively impair neuronal function. There is growing evidence that aging promotes insulin‐like growth factor 1 (IGF‐1) deficiency, which compromises multiple aspects of cerebromicrovascular and brain health. To determine the role of IGF‐1 deficiency in the pathogenesis of CMHs, we induced hypertension in mice with liver‐specific knockdown of IGF‐1 (*Igf1* ^*f/f*^ + TBG‐Cre‐AAV8) and control mice by angiotensin II plus l‐NAME treatment. In IGF‐1‐deficient mice, the same level of hypertension led to significantly earlier onset and increased incidence and neurological consequences of CMHs, as compared to control mice, as shown by neurological examination, gait analysis, and histological assessment of CMHs in serial brain sections. Previous studies showed that in aging, increased oxidative stress‐mediated matrix metalloprotease (MMP) activation importantly contributes to the pathogenesis of CMHs. Thus, it is significant that hypertension‐induced cerebrovascular oxidative stress and MMP activation were increased in IGF‐1‐deficient mice. We found that IGF‐1 deficiency impaired hypertension‐induced adaptive media hypertrophy and extracellular matrix remodeling, which together with the increased MMP activation likely also contributes to increased fragility of intracerebral arterioles. Collectively, IGF‐1 deficiency promotes the pathogenesis of CMHs, mimicking the aging phenotype, which likely contribute to its deleterious effect on cognitive function. Therapeutic strategies that upregulate IGF‐1 signaling in the cerebral vessels and/or reduce microvascular oxidative stress, and MMP activation may be useful for the prevention of CMHs, protecting cognitive function in high‐risk elderly patients.

## Introduction

Recent advances in magnetic resonance imaging techniques (e.g., T2* gradient recall echo and Susceptibility‐Weighted Imaging MRI sequences) have allowed the detection of previously undetectable small intracerebral hemorrhages, termed cerebral microhemorrhages (CMHs), in elderly patients. CMHs are typically regarded as small (<5 mm) vascular lesions associated with rupture of small intracerebral vessels and are considered of emerging importance as a contributing factor to the progressive impairment of neuronal function in aging (Poels *et al*. 2012; Akoudad *et al*. 2016). Clinical and experimental evidence confirm that the presence of CMHs is associated with decreases in processing speed and cognitive function (Poels *et al*. 2012; Akoudad *et al*. 2016), and manifestation of gait disturbances (de Laat *et al*. 2011; Choi *et al*. 2012; Toth *et al*. 2015b).

Epidemiological studies determined that aging and hypertension are the main risk factors for the development of cerebral microhemorrhages (CMHs) (Poels *et al*., 2011). Accordingly, the prevalence of CMHs significantly increases with age, from ~6.5% in persons aged 45 to 50 years to ~35 to 50% or more in elderly patients (Poels *et al*., 2011). In the Rotterdam Scan Study (Poels *et al*., 2011) in elderly patients, hypertension was a particularly strong risk factor for CMHs with a hazard ratio of 1.66. Other studies reached the same conclusion, reporting 56% prevalence for CMHs in elderly hypertensive subjects (Kato *et al*., 2002). Recent data from animal models extend the clinical findings, demonstrating that aging and hypertension synergistically interact to exacerbate the development of CMHs (Toth *et al*., 2015b). Our current understanding, based on experimental data, is that aging promotes the development of CMHs by exacerbating hypertension‐induced oxidative stress and redox‐sensitive activation of matrix metalloproteases (MMPs) compromising the structural integrity of the cerebral microvasculature (Toth *et al*., 2015b). Yet, the specific age‐related mechanism(s) that underlie the increased susceptibility of the aged cerebral vasculature to rupture remain elusive.

Work from our laboratories and others adds to the rapidly growing body of knowledge regarding the crucial role of endocrine mechanisms in age‐related cerebromicrovascular pathology. Particularly, the dramatic age‐related decline in circulating levels of insulin‐like growth factor 1 (IGF‐1) has been implicated in microvascular aging and cognitive decline (reviewed recently in Sonntag *et al*., 2013). IGF‐1 is a pleiotropic growth factor that possesses multifaceted vasoprotective effects. Epidemiological evidence suggests that low IGF‐1 levels increase the risk for cerebromicrovascular diseases (Sonntag *et al*., 2013). Previous research demonstrated that rodent models with decreases of circulating IGF‐1 levels also exhibit aging‐like vascular phenotypes including cerebromicrovascular autoregulatory dysfunction, manifestation of intracerebral hemorrhages, neurovascular uncoupling, increased vascular oxidative stress, dysregulation of genes encoding the ECM, and impaired functional adaptation of cerebral arteries to hypertension (Bailey‐Downs *et al*., 2012; Sonntag *et al*., 2013; Toth *et al*., 2014, 2015a; Tarantini *et al*., 2016a,b). Despite these advances, the pathophysiological link between age‐related circulating IGF‐1 deficiency and the development of CMHs has never been studied.

This study was designed to test the hypothesis that circulating IGF‐1 deficiency promotes the development of CMHs, by exacerbating hypertension‐induced vascular oxidative stress and MMP activation and/or by impairing microvascular structural adaptation to hypertension. To test our hypothesis, we used a novel mouse model of isolated endocrine IGF‐1 deficiency induced by adeno‐associated viral knockdown of IGF‐1 specifically in the mouse liver using Cre‐lox technology (*Igf1* ^*f/f*^ + TBG‐Cre‐AAV8; Toth *et al*., 2014, 2015a; Tarantini *et al*., 2016a,b). We induced hypertension in IGF‐1‐deficient mice and respective controls (by treatment with angiotensin II [Ang II] and the NO synthesis inhibitor l‐NAME) and compared the incidence, size, and localization of CMHs. To elucidate the mechanisms contributing to the changes in CMH incidence, hypertension‐induced vascular ROS production and MMP activation were assessed and hypertension‐induced microvascular remodeling and its molecular signature were quantified.

## Results

### Aging exacerbates hypertension‐induced spontaneous CMHs in mice

**Figure 1:**
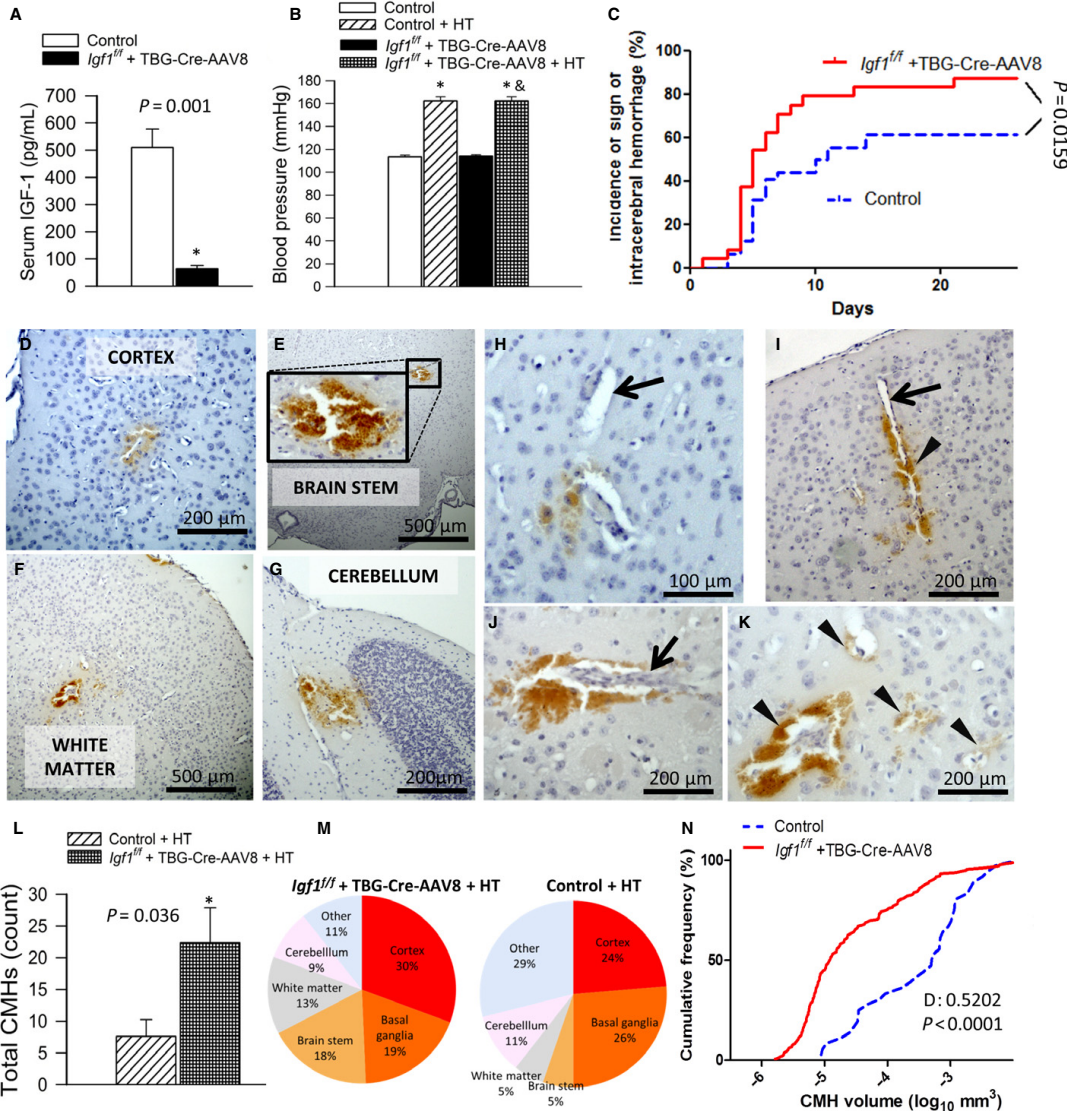
IGF‐1 deficiency exacerbates hypertension (HT)‐induced CMHs in mice. (A) Adeno‐associated viral knockdown of hepatic *Ifg1* (*Igf1* ^*f/f*^ + TBG‐Cre‐AAV8) significantly decreases the levels of circulating IGF‐1 compared to control animals. Data are mean ± SEM. *P = 0.001 vs. control (*t*‐test). (B) Treatment with angiotensin II plus l‐NAME elicited similar increases in systolic blood pressure in control and IGF‐1‐deficient mice. *P < 0.05 vs. control normotensive (one‐way ANOVA, Tukey's *post hoc* test). (C) Cumulative incidence curves for neurological signs of hypertension‐induced intracerebral hemorrhage in control (*n* = 24) and IGF‐1‐deficient mice (*n* = 31). In IGF‐1‐deficient mice, there was a significant increase in CMH incidence compared to control mice (log‐rank test; Mantel‐Cox). (D–G) Representative images of CMHs stained by diaminobenzidine in the cortex, brain stem, white matter, and cerebellum of hypertensive IGF‐1‐deficient mice. (H–K) Black arrows point to cerebral intraparenchymal arterioles in close proximity to the hemorrhages. Hemorrhages were often confined to and spread along the perivascular spaces (arrowheads). Note in (K) the spread of the hemorrhage to the daughter branches of an arteriole along the perivascular spaces. (L): Total number of hypertension‐induced CMHs throughout the entire brain of control and IGF‐1‐deficient mice. Data are mean ± SEM. *P < 0.05 (*t*‐test). (M) The pie charts illustrate the similar distribution of CMHs by location in both experimental groups. (N) The cumulative frequency distribution of CMHs by volume significantly shifts to the left in IGF‐1‐deficient mice compared to control, indicating that IGF‐1 deficiency specifically increases the number of smaller bleeds. The maximum difference between the cumulative distributions was calculated using the two‐sample Kolmogorov–Smirnov test (D: 0.5202; *P* < 0.0001).

Mice receiving TBG‐Cre‐AAV8 had significantly lower serum IGF‐1 levels compared with control mice receiving TBG‐eGFP‐AAV8 (Fig. 1A). Treatment with Ang II plus l‐NAME resulted in comparable increases in blood pressure both IGF‐1‐deficient mice and their age‐matched controls (Fig. 1B). We found that during the experimental period, 56% of control mice showed clinically manifest signs of hypertension‐induced intracerebral hemorrhage, as assessed by neurological examination. In contrast, 87% of IGF‐1‐deficient mice developed signs of hypertension‐induced intracerebral hemorrhage (Fig. 1C), which occurred within a similar time window (the maximum difference between the cumulative distribution curves for time‐to‐event in the two groups, D, is as follows: 0.2063; *P* = 0.753). Histological analysis confirmed that all mice with neurological signs developed multiple CMHs that were distributed widely in the brain (Fig. 1D–G). When the cerebral vessels associated with the CMHs were clearly distinguishable, their internal diameter was found to be in the range of ~10–20 μm (Fig. 1H–K). We noticed that the hemorrhages were often confined to and spread along the perivascular spaces (Fig. 1H–K). No normotensive mice developed neurological signs or histologically detectable CMHs (data not shown). In hypertensive IGF‐1‐deficient mice, a higher count of CMHs was observed compared to hypertensive mice with unaltered IGF‐1 levels (Fig. 1L). As shown in Fig. 1(M), IGF‐1 deficiency predominantly increased the incidence of CMHs located in the cortex, basal ganglia, brain stem, and white matter. Analysis of the volume distribution of CMHs (Fig. 1N) suggests that IGF‐1 deficiency significantly increases the incidence of the smallest hemorrhages, which originate from the distal portion of the microcirculation.

### Increased incidence of CMHs is associated with early gait dysfunction in IGF‐1‐deficient mice

**Figure 2:**
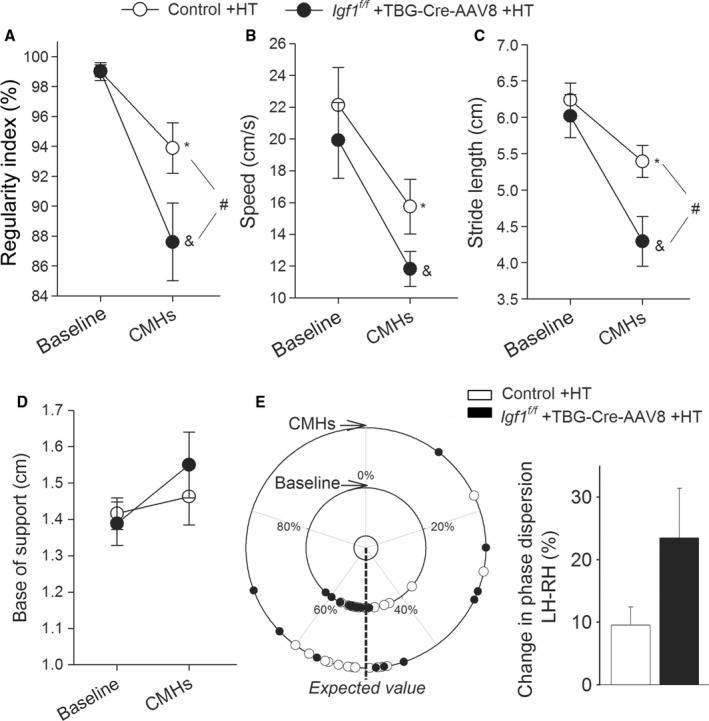
Increased incidence of CMHs is associated with gait dysfunction in hypertensive IGF‐1‐deficient mice. Regularity index (A), body speed (B), stride length (C), and base of support (front paws; D) in control mice and IGF‐1‐deficient mice under baseline conditions and after induction of CMHs. (E) Circular scatter plot showing the distribution of interlimb coupling values (phase dispersion) in control mice and IGF‐1‐deficient mice under baseline conditions and after induction of CMHs (note that the circular plot shows a smaller phase dispersion scatter in the inner circle [before hypertension] as compared to the phase dispersion scatter in the outer circle [assessed after hypertension]). Right panels shows average deviation of phase dispersion (calculated between the right hind paw [RH] and left hind paw [LH]) from the expected value (50%) under baseline conditions and after induction of CMHs. Data are mean ± SEM.*P < 0.05 vs. control baseline, ^&^ *P* < 0.05 vs. IGF‐1‐deficient baseline, ^#^ *P* < 0.05 control vs. IGF‐1 deficient (one‐way ANOVA, Tukey's *post hoc* test) HT: hypertension.

As in humans CMHs are associated with gait dysfunction (Choi *et al*., 2012), we analyzed mouse gait. We found that gait abnormalities (including a significant decline in regularity index, speed, and stride length; Fig. 2A–C, respectively) were significantly more severe in hypertensive IGF‐1‐deficient mice as compared to hypertensive control mice, suggesting that analysis of motor function status (e.g., deficit in interlimb coordination, temporal asymmetry) can predict the severity of CMH burden (Toth *et al*., 2015b). Hypertension‐induced changes in base of support did not correlate with IGF‐1 status (Fig. 2D). Phase dispersion tended to change more in hypertensive IGF‐1‐deficient mice as compared to hypertensive controls, but the difference did not reach statistical significance (Fig. 2E). Gait parameters in normotensive IGF‐1‐deficient mice and age‐matched control mice did not change significantly during the experimental period (data not shown).

### IGF‐1 deficiency exacerbates hypertension‐induced MMP activation

**Figure 3:**
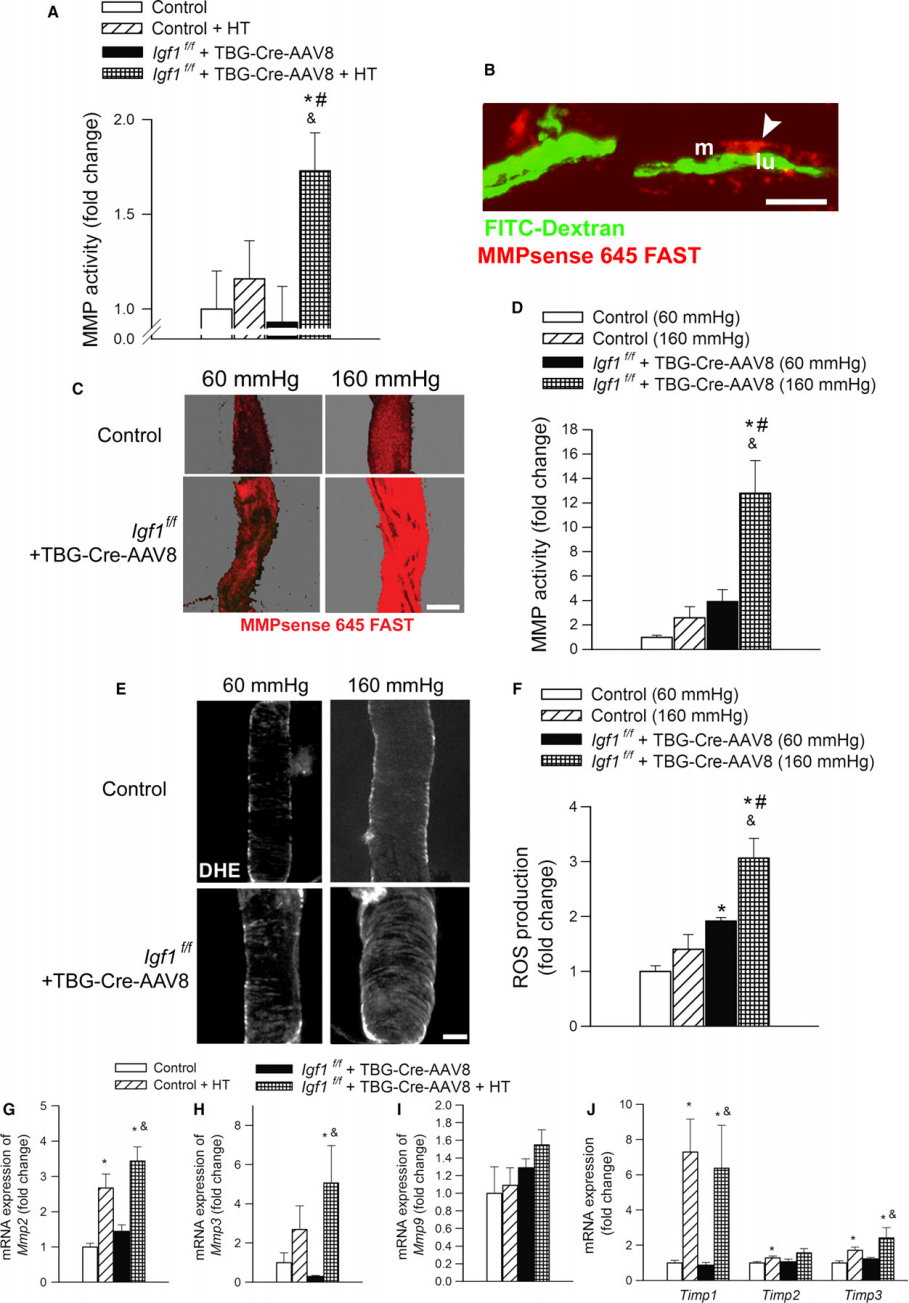
IGF‐1 deficiency exacerbates hypertension‐induced MMP activation. (A) Hypertension‐induced MMP activation, assessed using the MMPsense 645 FAST fluorescent method, in control and IGF‐1‐deficient mice (*n* = 6 in each group; see Experimental procedures). MMPsense 645 FAST becomes fluorescent upon cleavage by activated MMPs. Data are mean ± SEM. *P < 0.05 vs. control, ^#^ *P* < 0.05 vs. control HT, ^&^ *P* < 0.05 vs. IGF‐1 deficient. (B) Representative confocal image of the longitudinal section of a cerebral intraparenchymal arteriole from a hypertensive IGF‐1‐deficient mouse injected with the MMPsense 645 FAST substrate (scale bar: 25 μm). Note, the strong red fluorescence in the vascular wall indicating increased MMP activity. Intraluminal FITC–dextran is shown for orientation purposes. L (lumen), M (media). (C): Representative compressed Z stacks of confocal images of MCAs showing stronger MMPsense 645 FAST fluorescence (red) in high‐pressure‐exposed MCAs isolated from IGF‐1‐deficient mice as compared to MCAs isolated from control mice, indicating increased MMP activation. MCAs were pressurized at 60 and 160 mmHg for 6 h. (original magnification: 20×, scale bar: 100 μm). Bar graphs (D) are summary data. Data are means ± SEM (*n* = 6 in each group) *P < 0.05 vs. control (60 mmHg), ^#^ *P* < 0.05 vs. IGF‐1 deficient (160 mmHg); ^&^ *P* < 0.05 vs. IGF‐1 deficient (60 mmHg). (E) IGF‐1 deficiency exacerbates hypertension‐induced oxidative stress. Representative confocal images showing stronger DHE fluorescence (pseudocolored white) indicating increased $O_{2}^{\cdot -}$ production in high‐pressure‐exposed MCAs isolated from aged mice as compared to MCAs isolated from young mice. MCAs were pressurized at 60 and 160 mmHg for 6 h. (original magnification: 20×, scale bar: 50 μm). Bar graphs (F) are summary data. Data are means ± SEM (*n* = 6 in each group). *P < 0.05 vs. control, ^#^ *P* < 0.05 vs. control HT, ^&^ *P* < 0.05 vs. IGF‐1 deficient. G–J show hypertension‐induced changes in mRNA expression of MMP‐2, ‐3, and ‐9 as well as Timp‐1, ‐2, and ‐3 in the cerebral arteries. Data are mean ± SEM (*n* = 6 in each group). *P < 0.05 vs. control, ^&^ *P* < 0.05 vs. IGF‐1 deficient. Differences between different groups were established using a one‐way ANOVA followed by Tukey's *post hoc* tests.

Hypertension‐induced activation of MMPs is thought to play a central role in the pathogenesis of CMHs (Wakisaka *et al*., 2010a,b; Toth *et al*., 2015b). To determine how interaction of IGF‐1 deficiency and hypertension affect vascular MMP activity, MMPsense 645 FAST substrate was administered *in vivo* and vascular MMP activation was compared between hypertensive IGF‐1‐deficient mice and hypertensive age‐matched controls by measuring MMPsense fluorescence in brain homogenates. Figure 3(A) shows that the brains of hypertensive IGF‐1‐deficient mice exhibit significantly greater MMP activity compared to those of the normotensive counterparts. Mice with normal IGF‐1 levels did not show a significant hypertension‐induced increase in MMP activity. In control mice, MMP activity (indicated by the presence of the fluorescent product of the MMPsense 645 FAST substrate) was barely detectable by confocal microscopy and was not increased significantly by hypertension. The representative image, shown in Fig. 3(B), illustrates that in IGF‐1‐deficient mice, hypertension was associated with strong MMPsense 645 FAST fluorescence (indicating MMP activation) localized mainly to the media of small intracerebral arteries (identified by the intraluminal FITC–dextran fluorescence). No significant MMP 645 FAST fluorescence was observed in the brain parenchyma in any of the groups. Collectively, these findings suggest that IGF‐1 deficiency exacerbates hypertension‐induced vascular MMP activation.

Results of previous experimental studies by our laboratories (Toth *et al*., 2015b), and by others (Wakisaka *et al*., 2010a,b), identify oxidative stress as a critical factor contributing to hypertension‐induced MMP activation and pathogenesis of CMHs and demonstrate that high intraluminal pressure *per se* (via increased wall tension‐dependent cellular stretch) is a key stimulus for increased vascular production of ROS (Ungvari *et al*., 2003; Springo *et al*., 2015; Toth *et al*., 2015b) that lead to MMP activation. To elucidate the likely mechanism contributing to the exacerbation of hypertension‐induced MMP activation in IGF‐1 deficiency, we compared pressure‐induced production of $O_{2}^{\cdot -}$ in cerebral arteries isolated from IGF‐1‐deficient mice and their respective age‐matched controls using the redox‐sensitive dye dihydroethidium (DHE). We found that nuclear DHE fluorescence was significantly stronger in arteries of IGF‐1‐deficient mice that were exposed to high pressure as compared to vessels of control mice exposed to the same pressure or vessels of the same IGF‐1‐deficient animals that were exposed to 60 mmHg (Fig. 3E,F). The findings that IGF‐1 deficiency exacerbates high‐pressure increases in $O_{2}^{\cdot -}$ generation in the cerebral arteries are significant as high‐pressure‐induced vascular MMP activation can be inhibited by antioxidant treatments (Toth *et al*., 2015b).

We also detected and analyzed the mRNA expression of MMPs and the tissue inhibitor of metalloproteinases (TIMPs; Fig. 3G–J). We found that the expression of *Mmp2*,* Mmp3* but not *Mmp9* increased significantly in the brains of hypertensive IGF‐1‐deficient mice when compared to their normotensive counterpart. The expression of *Timp1*,* Timp2*, and *Timp3* consistently increased in response to hypertension in all experimental groups, except Timp2 mRNA expression did not reach significance in hypertensive IGF‐1‐deficient animals when compared to their normotensive counterpart.

### IGF‐1 deficiency impairs structural adaptation of cerebral arterioles to hypertension

**Figure 4:**
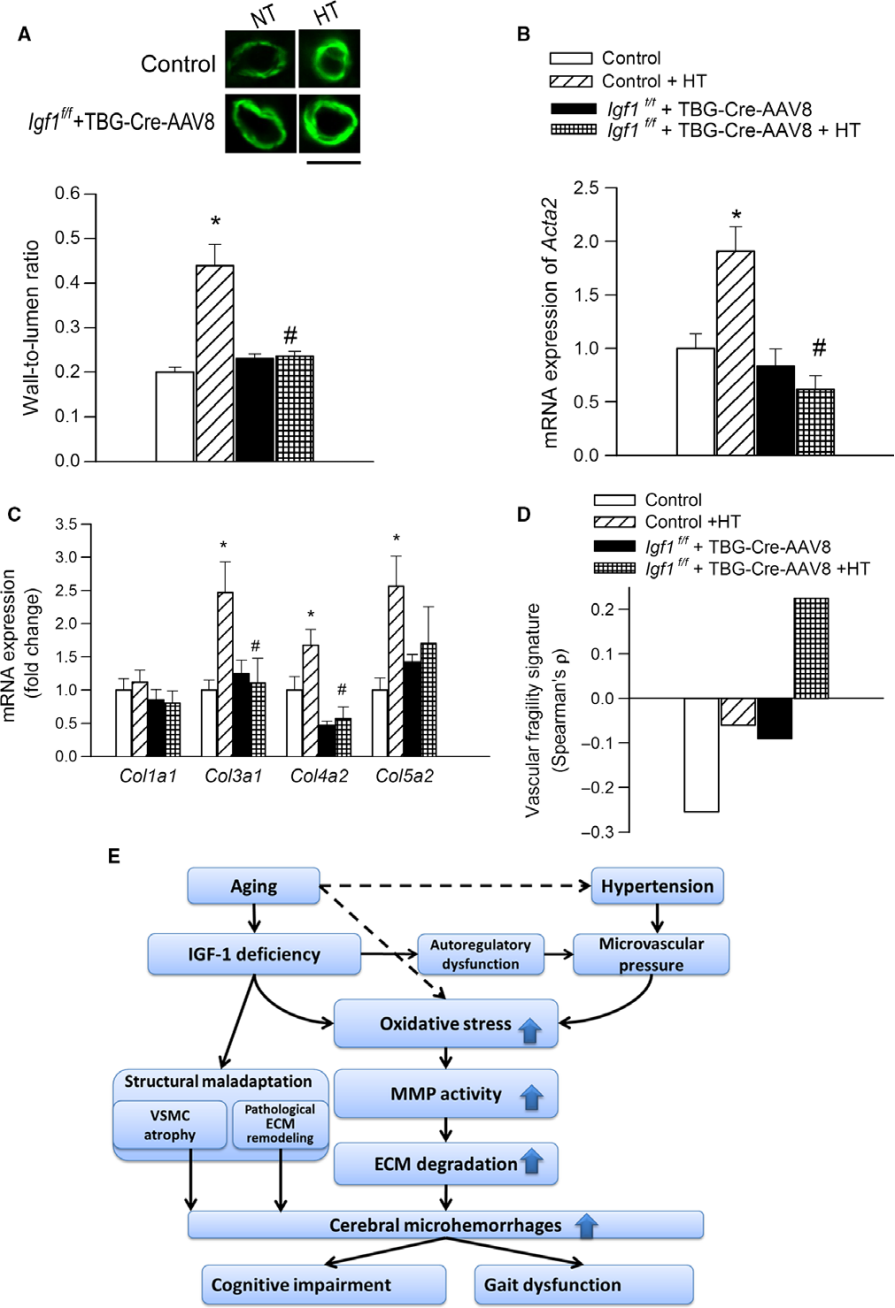
IGF‐1 deficiency impairs hypertension‐induced hypertrophy and structural remodeling in cerebral vessels. (A) Representative confocal micrographs from normotensive (NT) and hypertensive (HT) fixed brains. Hypertension induces hypertrophy of penetrating arterioles in control mice, whereas this adaptive response is impaired in IGF‐1‐deficient mice (green fluorescence: immunostaining for alpha smooth muscle actin). Bar graphs are summary data for calculated wall‐to‐lumen ratios. qPCR data showing mRNA expression of alpha smooth muscle actin and collagens in branches of the middle cerebral arteries isolated from normotensive and hypertensive control and IGF‐1‐deficient mice are shown in panels B and C, respectively. Data are mean ± SEM (*n* = 4–6 in each group), *P < 0.05 vs. control, ^#^ *P* < 0.05 vs. control HT. (D) IGF‐1 deficiency exacerbates hypertension‐induced profragility shift in vascular gene expression signature. Expression of 67 genes related to the pathogenesis of CMHs was determined by qPCR, and vascular fragility signatures (Spearman's ρ) were calculated as described in the Results. A higher ‘fragility signature’ indicates higher expression of profragility genes and lower expression of antifragility regulators. (E) Proposed scheme depicting the mechanisms by which age‐related IGF‐1 deficiency may exacerbate hypertension‐induced microvascular damage, promoting CMHs. Differences between different groups were established using a one‐way ANOVA followed by Tukey's *post hoc* tests.

Media hypertrophy and extracellular matrix remodeling are adaptive processes that reduce tensile stress, protecting the integrity of the vascular wall in hypertension. We found that penetrating arterioles of control mice exhibited structural adaptation to high pressure (manifested as a significant increase in wall‐to‐lumen ratios; Fig. 4A), which was associated with upregulated vascular expression of alpha smooth muscle actin and components of the ECM (Fig. 4B,C). In contrast, histological and molecular signs of protective structural adaptation of cerebral vessels were not evident in hypertensive IGF‐1‐deficient mice (Fig. 4A–C).

### IGF‐1 deficiency exacerbates hypertension‐induced profragility shift in vascular gene expression signature

Expression of genes related to the pathogenesis of CMHs was determined by qPCR; 67 genes analyzed (Table S1) were manually annotated as being provascular fragility and antifragility, and this data were converted into a binary vector (1 for profragility genes and −1 for antifragility genes). qPCR data were normalized using quantile normalization, and then, for each sample, the binary fragility vector was compared to negative normalized Ct values using Spearman's rank correlation coefficient (Spearman's ρ) as reported (Tarantini *et al*., 2016b). This ‘vascular fragility signature’, therefore, measures the combined expression levels of positive and negative regulators of vascular fragility: a higher value indicates higher expression of profragility genes and lower expression of antifragility genes. We found that IGF‐1 deficiency exacerbates hypertension‐induced profragility shift in vascular gene expression signature (Fig. 4D).

## Discussion

This is the first study to demonstrate that the effects of IGF‐1 deficiency phenocopies important aspects of age‐related CMHs (Toth *et al*., 2015b). Similar to the aging phenotype, in IGF‐1 deficiency, the same level of hypertension leads to significantly increased incidence of CMHs. In human patients, hypertension also almost exclusively increases CMHs at an old age (Romero *et al*., 2014). We found that similar to aging, IGF‐1 deficiency predominantly increases the incidence of small cerebral hemorrhages in a cortical/subcortical location, suggesting that they both render the same small cerebral vessels significantly more vulnerable to high‐pressure‐induced rupture.

Circulating IGF‐1 levels significantly decline with age (reviewed in Sonntag *et al*., 2013). Our findings underscore the likely pathogenic role of age‐related IGF‐1 deficiency in increased CMHs in elderly patients. Indeed, there are many similarities between CMHs observed in IGF‐1‐deficient mice and hypertensive elderly human patients, including the relative size of the bleedings, the clinical symptoms, and the progressive nature of the pathological process (Lee *et al*., 2011).

Clinical studies show that CMHs are an important mechanism for cognitive impairment (Seo *et al*., 2007), and their increased prevalence and incidence in aging are consistent with the documented age‐related exacerbation of hypertension‐induced cognitive decline in humans and laboratory animals. CMHs have also been shown to be associated with gait dysfunction both in humans (Choi *et al*., 2012) and experimental animals. Importantly, we also revealed that increased incidence of CMHs leads to progressive gait abnormalities in IGF‐1‐deficient mice mimicking the phenotype observed in hypertensive aged mice (Toth *et al*., 2015b). Gait is a complex motor behavior which involves several brain regions that coordinate to produce locomotion. Of the aforementioned studies, the view emerges that gait abnormalities are sensitive indicators of CMHs as lesions affecting any brain region important for gait coordination (e.g., multiple cortical areas, basal ganglia, cerebellum, and white matter) will elicit quantifiable symptoms. The causal link between CMHs and gait abnormalities is clinically potentially significant as gait impairments are a major risk factor for falls in the elderly. Further, increasing clinical evidence shows that gait and cognition are interrelated in older adults (Montero‐Odasso *et al*., 2012).

The IGF‐1‐dependent mechanisms responsible for increased susceptibility of the cerebral circulation to hypertension‐induced injury are likely multifaceted. Previous studies suggest a central role for age‐related exacerbation of hypertension‐induced cerebrovascular ROS production and redox‐sensitive activation of MMPs in the pathogenesis of CMHs (Toth *et al*., 2015b), which degrade components of the basal lamina and extracellular matrix, weakening the vascular wall. Our findings suggest that IGF‐1 deficiency itself can exacerbate hypertension‐induced cerebrovascular MMP activation, mimicking the aging phenotype, which likely importantly contribute to the increased fragility of cerebral arteries. While IGF‐1 deficiency does not appear to be associated with marked changes in MMP expression, it was found to exacerbate hypertension‐induced oxidative stress in cerebral arteries, presenting redox‐sensitive MMP activation (Wakisaka *et al*., 2010a) as a potential mechanism responsible for the observed phenotype. This concept is supported by recent findings showing that age‐related exacerbation of hypertension‐induced MMP activation can be abolished by antioxidative treatments (Toth *et al*., 2015b). Increased hypertension‐induced oxidative stress in aged arteries has been attributed to upregulation of NOX oxidases, increased mitochondrial ROS generation, and impaired Nrf2‐dependent antioxidant defense mechanisms (Ungvari *et al*., 2011a,b; Springo *et al*., 2015; Toth *et al*., 2015b). There is evidence that inhibition of ROS synthesis by these sources can prevent development of CMHs in aging (Toth *et al*., 2015b). The existing evidence suggest that circulating IGF‐1 deficiency affects the same redox pathways as aging (Csiszar *et al*., 2008; Bailey‐Downs *et al*., 2012). Thus, future studies are warranted to determine the effects of inhibitors of NOX oxidases and mitochondria‐derived ROS production on the genesis of CMHs in IGF‐1 deficiency as well. We posit that development of CMHs in the elderly and in IGF‐1‐deficient patients may also be exacerbated by deficiency of NO due to its increased breakdown by elevated levels of ROS (Toth *et al*., 2015a). NO deficiency likely amplifies the effect of angiotensin II and significantly increases the stiffness of the conduit arteries, impairing Windkessel function, increasing pulse pressure, and promoting penetration of the pressure wave into the cerebral microcirculation (Tarumi *et al*., 2014).

The mechanisms by which IGF‐1 deficiency promote CMHs likely also involve impaired structural and functional adaptation of the cerebral circulation to hypertension. Several lines of evidence support this concept. During hypertension, healthy cerebral arterioles undergo structural remodeling including hypertrophy of the media (Baumbach & Heistad, 1989), which reduce circumferential stress, preventing mechanical damage to the vascular wall. Our findings suggest that IGF‐1 deficiency perturbs arteriolar remodeling processes by impairing hypertension‐induced adaptive media hypertrophy and extracellular matrix remodeling. It is likely that the resulting increases in circumferential stress are causally linked to the increased susceptibility to CMHs. High‐pressure/increased wall stress itself appears to be the main stimulus for increased vascular MMP activation, as well as ROS production associated with IGF‐1 deficiency and aging (Toth *et al*., 2015b). Thus, it is likely that penetration of increased arterial pressure to the vulnerable distal portion of the cerebral microcirculation is a critical factor in the development of CMHs. In support of this concept, previous studies demonstrated that aging results in autoregulatory dysfunction both in human patients and experimental animals (Toth *et al*., 2015b), likely contributing the pathogenesis of CMHs. In that regard, it is significant that IGF‐1 deficiency is also associated with cerebral autoregulatory dysfunction and impaired myogenic adaptation of the cerebral resistance arteries to high pressure (Toth *et al*., 2014), which likely allows sudden increases in blood pressure to inflict damage to the thin‐walled cerebral microvessels.

In conclusion, our results add to the growing evidence that circulating IGF‐1 exerts complex cerebromicrovascular protective effects and that cerebromicrovascular dysfunction associated with age‐related IGF‐1 deficiency compromises multiple aspects of brain health (Sonntag *et al*., 2013). The findings that IGF‐1 deficiency promotes the pathogenesis of CMHs by exacerbating hypertension‐induced cerebrovascular oxidative stress and MMP activation and leading to structural and functional (Toth *et al*., 2014) maladaptation to hypertension (Fig. 4E) have important clinical relevance for the pathogenesis of vascular cognitive impairment and gait abnormalities in elderly hypertensive patients. Our findings, taken together with the results of earlier studies (reviewed in Sonntag *et al*., 2013), point to potential benefits of interventions preventing age‐related decline in circulating IGF‐1 levels and promoting microvascular health for prevention of CMHs and cognitive decline in the elderly. Although treatment with recombinant IGF‐1 in the elderly is currently not recommended due to a potentially increased risk for cancer, other treatment options that increase vascular IGF‐1 signaling, attenuate vascular ROS production, and/or reduce MMP activation could be considered for cerebromicrovascular protection in older individuals at risk for CMHs.

## Experimental procedures

All procedures were approved by and followed the guidelines of the Institutional Animal Care and Use Committee of the University of Oklahoma HSC and are reported in accordance with the ARRIVE guidelines.

### Induction of adult‐onset IGF‐1 deficiency in mice

Male mice homozygous for a floxed exon 4 of the *Igf1* gene (*Igf1* ^*f/f*^; in a C57BL/6 background) were purchased from Jackson Laboratories (Bar Harbor, ME, USA). These mice have the entirety of exon 4 of the *Igf1* gene flanked by loxP sites, which allows for genomic excision of this exon when exposed to Cre recombinase. Transcripts of the altered *Igf1* gene yield a protein upon translation that fails to bind the IGF receptor. Adult‐onset circulating IGF‐1 deficiency was induced in *Igf1* ^*f/f*^ mice by adeno‐associated virus (AAV8)‐mediated expression of Cre recombinase in the liver at 4 months of age, as reported (Toth *et al*., 2014). The AAV8 vector was purchased from the University of Pennsylvania Viral Vector Core (Penn Vector Core, Philadelphia, PA, USA; http://www.med.upenn.edu/gtp/vectorcore). Although AAV8 is effective at transducing multiple tissues, the use of thyroxine‐binding globulin (TBG) promoter allows for the restriction of expression to hepatocytes. At 4 months of age, *Igf1* ^*f/f*^ mice were randomly assigned to two groups and were administered approximately 1.3 × 10^10^ viral particles of AAV8‐TBG‐Cre or AAV8‐TBG‐eGFP via retro‐orbital injection, as described. Circulating IGF‐1 is produced in the liver. As circulating IGF‐1 levels during adolescence play a critical role in development of many organs, including the cardiovascular system, this mouse model was developed to be able to selectively study the consequences of adult‐onset circulating IGF‐1 deficiency (Toth *et al*., 2014). Animals were housed in the Rodent Barrier Facility at OUHSC under specific pathogen‐free barrier conditions, on a 12‐h light/12‐h dark cycle, with access to standard rodent chow (Purina Mills, Richmond, IN, USA) and water *ad libitum*.

### Measurement of serum IGF‐1 levels

Submandibular venous blood was collected into microcentrifuge tubes using a sterile lancet (Medipoint, Mineola, NY, USA) according to the manufacturer's instructions. Whole blood was centrifuged at 2500 *g* for 20 min at 4 °C to collect serum, which was then stored at −80 °C. IGF‐1 concentration in the serum samples was measured by ELISA (R&D Systems, Minneapolis, MN, USA) as reported (Toth *et al*., 2014). An IGF‐1 control sample, with aliquots stored at −80 °C, was included on each plate. Serum IGF‐1 levels are reported in ng mL^−1^.

### Induction of spontaneous CMHs

To study the effects of IGF‐1 deficiency on spontaneous, hypertension‐induced CMHs, we used a previously well‐characterized mouse model (Toth *et al*., 2015b). Briefly, in 10‐month‐old male IGF‐1‐deficient mice (*Igf1* ^*f/f*^ + TBG‐Cre‐AAV, *n *=* *60) and respective age‐matched control mice (*Igf1* ^*f/f* ^+ TBG‐eGFP‐AAV8, *n *=* *60), hypertension was induced by a combination treatment with ω‐nitro‐l‐arginine‐methyl ether (l‐NAME, 100 mg kg^−1^ day^−1^, in drinking water) and administration of angiotensin II (Ang II; s.c. via osmotic mini‐pumps [Alzet Model 2006, 0.15 μL h^−1^, 42 days; Durect Co, Cupertino, CA, USA]). Pumps were filled either with saline or solutions of angiotensin II (Sigma Chemical Co., St. Louis, MO, USA) that delivered (subcutaneously) 1 μg min^−1 ^kg^−1^ of angiotensin II for 28 days, thus generating four experimental groups: (1) *Igf1* ^*f/f *^+ TBG‐Cre‐AAV8 + Ang II, (2) *Igf1* ^*f/f*^ + TBG‐Cre‐AAV8 + vehicle, (3) *Igf1* ^*f/f*^ + TBG‐eGFP‐AAV8 + Ang II, and (4) *Igf1* ^*f/f*^ + TBG‐eGFP‐AAV8 + vehicle. Pumps were placed into the subcutaneous space of ketamine/xylazine anesthetized mice through a small incision in the interscapular area that was closed with surgical sutures using aseptic techniques. All incision sites healed rapidly without the need for additional medication. As aging is associated with increased activity of the vascular renin–angiotensin system and Ang II‐dependent hypertension is common among older individuals, Ang II‐dependent hypertension is a clinically highly relevant model to study aging‐related cerebrovascular alterations (Toth *et al*., 2013).

Blood pressure of the animals was recorded before the treatment and every second day during the treatment period using a tail‐cuff blood pressure apparatus (CODA NonInvasive Blood Pressure System; Kent Scientific Co., Torrington, CT, USA), as described (Toth *et al*., 2015b). Each experimental group was closely monitored and mice were killed upon the occurrence of neurological signs of intracerebral hemorrhages. For cross‐sectional studies (including histology, MMP activation, molecular studies), a second cohort of animals was sacrificed on day 10 postinduction of hypertension.

### Standardized neurological examination of mice

To assess the occurrence of clinically manifest hemorrhages daily, neurological examination was performed as reported (Toth *et al*., 2015b), by assessing each animal's spontaneous activity, symmetry in the movement of the four limbs, forelimb outstretching, climbing ability, body proprioception, response to vibrissae touch, and gait coordination. Each examined animal was provided with a daily score calculated by the summation of all individual test scores. When a consistent decline in the neurological score was observed or on day 28 of the study, mice were euthanized by CO_2_ asphyxiation.

### Analysis of gait function

To assess gait function and the spatial and temporal aspects of interlimb coordination, the animals were tested using the CatWalk System (Noldus Information Technology Inc., Leesburg, VA, USA), as reported (Toth *et al*., 2015b). Briefly, animals were trained to cross the walkway and then, in a dark room, had five consecutive volunteer runs on the day before and on each day after induction of hypertension. CMH‐related changes in speed, base of support, interpaw phase dispersion/phase lag, stride length, and regularity index were assessed. The regularity index (%) is a fractional measure of interpaw coordination, which expresses the number of normal step sequence patterns relative to the total number of paw placements. Its value in healthy animals is ~100%. Phase dispersion is a measure of the temporal relationship between placement of two hind paws within a step cycle.

### Histological analysis of intracerebral hemorrhages

Mice were euthanized and transcardially perfused with ice‐cold heparinized PBS for 5 min and subsequently decapitated as reported (Toth *et al*., 2015b). Then, the brains were isolated and fixed in 10% formalin at room temperature for one day. The next day, the brains were placed in fresh 10% formalin (at 4 °C, for 2 days), then in 70% ethanol (at 4 °C, for 2 days), followed by embedding in paraffin. The brains were serially sectioned at 8 μm thickness, yielding approximately 1500 sections per brain. The first two sections of every five section were stained with hematoxylin to reveal the brain structure and diaminobenzidine (DAB) to highlight the presence of hemorrhages. DAB turns into dark brown when it undergoes a reaction with peroxidases present in red blood cells, therefore allowing precise detection of extravasated blood cells in the parenchyma of the brain. All stained sections were screened by a reader blinded to the treatment groups, and images were acquired in the evidence of a positive DAB reaction. Digital images were analyzed with imagej software (NIH) to identify the location and quantify the number and size of hemorrhages. The volumetric reconstruction of hemorrhages was estimated according to the following formula as described (Toth *et al*., 2015b).

$$\text{CMH volume}\;\left({\text{mm}}^{3}\right)=\sum_{n=1}^{i}\left[3\times \text{(CMH}\;{\text{area)}}_{i}\times \text{(slice}\;{\text{thickness)}}_{i}\right].$$

### Assessment of hypertension‐induced MMP activation in the cerebral vessels *in situ*

Mice from each experimental group were temporarily anesthetized with ketamine/xylazine and injected retro‐orbitally with a 100 μL dose of 40 nmol L^−1^ MMPsense 645 FAST substrate (PerkinElmer Inc., Boston, MA, USA; 3 μmol L^−1^; at 37 °C, for 6 h, in the dark), as reported (Toth *et al*., 2015b). This substrate is normally optically inert. Once it is cleaved, its subunits become excitable at 649 nm and emit a red signal that can be measured as an indicator of activity of MMP 2, 3, 7, 9, 12, and 13. After 12 h of circulation of the substrate, animals were transcardially perfused with ice‐cold PBS containing 1 × heparin and FITC–dextran (to highlight the vascular lumen). Then, the mice were decapitated and the brains were removed and cut in half. From the left hemisphere, the frontal cortex containing the photoactive substrate was isolated and homogenized. To quantify MMP activity, the background‐corrected fluorescence (Ex: 649 nm, Em: 666 nm) was measured spectrophotofluorometrically using a microplate reader and normalized to tissue weight, as described (Toth *et al*., 2015b). The right hemisphere was embedded in OCT (optimum cutting temperature) media and cryosectioned, and confocal images of brain areas containing cross sections of penetrating small arteries were captured as described (Toth *et al*., 2015b).

### Detection of high‐pressure‐induced activation of MMPs in isolated cerebral arteries

In separate experiments, pressure‐induced MMP activity was measured in cannulated segments of the middle cerebral arteries, as described (Toth *et al*., 2015b). In brief, two segments of the middle cerebral arteries were isolated from the brains of mice from control and IGF‐1‐deficient mice. The vessels were mounted onto two glass micropipettes in an organ chamber in oxygenated (21% O_2_, 5% CO_2_, 75% N_2_) Krebs’ buffer (composed of [in mmol L^−1^]: 110.0 NaCl, 5.0 KCl, 2.5 CaCl_2_, 1.0 MgSO_4_, 1.0 KH_2_PO_4_, 5.5 glucose, and 24.0 NaHCO_3_, pH ~7.4; at 37 °C) and pressurized to 10 mmHg. Inflow and outflow pressures were controlled and measured by a pressure servo‐control system (Living Systems Instrumentation, Burlington, VE, USA). Vessels from the same animals were pressurized to 60 or 160 mmHg (to recapitulate *in vitro* the hemodynamic environment present in the vascular system during normotension and hypertension, respectively) in the presence of MMPsense 645 FAST substrate for 8 h. After the incubation period, the vessels were thoroughly rinsed, placed on a glass slide, and imaged with a Leica SP2 upright confocal microscope. The detected fluorescence intensity emitted at 666 nm was measured, corrected for the background, and normalized to vessel surface area using the metamorph software (Molecular Devices LLC, Sunnyvale, CA, USA).

### Detection of high‐pressure‐induced production of ROS in isolated cerebral arteries

In separate experiments, pressure‐induced ROS production was measured in cannulated segments of the middle cerebral arteries, as described (Springo *et al*., 2015; Toth *et al*., 2015b). In brief, the arteries from each control and IGF‐1‐deficient mice were pressurized to 60 or 160 mmHg for 4 h. To characterize high‐pressure‐induced vascular ROS production, at the end of the incubation period, the vessels were loaded with the redox‐sensitive dye DHE (Invitrogen, Carlsbad, CA, USA; 3 × 10^−6^ mol L^−1^; for 30 min). After loading, the chamber was washed out five times with warm Krebs buffer, and the vessels were allowed to equilibrate for another 20 min. After the experimental period, confocal images of the wall of the pressurized vessels were captured using a Leica SP2 confocal laser scanning microscope (Leica Microsystems GmbH, Wetzlar, Germany). Average nuclear DHE fluorescence intensities were assessed using the metamorph software (Molecular Devices LLC, Sunnyvale, CA, USA), and values for each animal in each group were averaged.

### Assessment of hypertension‐induced hypertrophy of penetrating arterioles

To determine the effect of IGF‐1 deficiency on adaptive hypertension‐induced hypertrophy of the arteriolar wall, on day 10 postinduction of hypertension, brains were perfusion‐fixed (4% ice‐cold paraformaldehyde; at 100 mmHg). Then, frozen OCT‐embedded sagittal sections (35 μm) were cut and stored free‐floating in cryoprotectant solution (25% glycerol, 25% ethylene glycol, 25% of 0.1 m phosphate buffer, and 25% water) at −20 °C. Sections were rinsed with Tris‐buffered saline (TBS), permeabilized with TBS with 0.05% Tween‐20. Antigen retrieval was achieved using 10 mm citrate buffer (10 mm sodium citrate and 0.05% Tween 20, pH 6.0) at 90 °C for 20 min followed by 1% sodium borohydride in PBS at room temperature for 30 min. After blocking with 5% BSA and 1% fish gelatin in TBS at room temperature for 2 h, sections were immunostained using rabbit anti‐alpha smooth muscle actin (ab5694 1:200; Abcam, Cambridge, MA, USA) primary antibody for 24 h at 4 °C. Sections were washed for 10 min in TBS (three times), incubated in Alexa Fluor 488 goat anti‐rabbit secondary antibody (A11070, 1:200; Life Technologies) for 1 h at room temperature, washed for 10 min (three times) in TBST, transferred to slides, and coverslipped. Confocal images of penetrating arterioles were obtained using Leica SP2 MP confocal laser scanning microscope. Wall‐to‐lumen ratios were calculated using the Metamorph software, and values for each animal were averaged.

### Determination of expression changes in CMH‐related genes by quantitative real‐time RT–PCR

To predict gene targets associated with vascular fragility and CMHs, we used the IRIDESCENT (Wren & Garner, 2004) text mining package, as described (Toth *et al*., 2015a,b). Using this method, important genes relevant for the pathogenesis of CMHs and structural integrity of the vasculature were identified (Table S1). The mRNA expression of these genes in cerebral arteries isolated on day 10 post‐induction of hypertension was analyzed by a quantitative real‐time RT–PCR technique using a Strategen MX3000 platform, as previously reported (Toth *et al*., 2015b). In brief, total RNA was isolated with a Mini RNA Isolation Kit (Zymo Research, Orange, CA, USA) and was reverse‐transcribed using Superscript III RT (Invitrogen). Amplification efficiencies were determined using a dilution series of a standard vascular sample. Quantification was performed using the efficiency‐corrected ΔΔCq method. The relative quantities of the reference genes *Hprt*,* Ywhaz*,* B2m*,* Gapdh*,* Actb*, and *S18* were determined, and a normalization factor was calculated based on the geometric mean for internal normalization. Fidelity of the PCR was determined by melting temperature analysis and visualization of the product on a 2% agarose gel.

### Statistical analysis

An *a priori* power analysis was performed to ensure 80% or greater power for the primary outcome measures, considering the findings of previous studies (Toth *et al*., 2015b). Cumulative incidence of signs of hemorrhage was evaluated using a Kaplan–Meier test, and the difference among groups was analyzed by log‐rank test (Mantel‐Cox). Differences between different groups were established using a one‐way ANOVA followed by Tukey's *post hoc* tests. *P* < 0.05 was considered significant. Data are expressed as mean ± SEM. Statistical analyses were conducted using prism 5 software (GraphPad, La Jolla, CA, USA).

## Funding

This work was supported by grants from the American Heart Association (to ST, MNVA, AC and ZU), the National Center for Complementary and Alternative Medicine (R01‐AT006526 to ZU), the National Institute on Aging (R01‐AG047879; R01‐AG038747; 3P30AG050911‐02S1), the National Institute of Neurological Disorders and Stroke (NINDS; R01‐NS056218 to AC), the National Heart, Lung and Blood Institute (1R01HL132553‐01), the Arkansas Claude Pepper Older Americans Independence Center at University of Arkansas Medical Center (to ZU; P30 AG028718), the Oklahoma Center for the Advancement of Science and Technology (to AC, ZU), the Oklahoma IDeA Network for Biomedical Research Excellence (to AC), the NIA‐supported Geroscience Training Program in Oklahoma (T32 AG052363‐01), and the Reynolds Foundation (to ZU and AC).

## Conflict of interest

None declared.

## Author contribution

ST, AC, and ZU designed research; ST, MNVA, AY, NA, GF, ZS, TG performed experiments; ST, ZS, MNVA, AC, CG, JW, WES, and ZU analyzed and interpreted data; ST, AC, and ZU wrote and revised the manuscript.

## Supporting information


**Table S1** List of important genes relevant for the structural integrity of the vasculature and potentially the pathogenesis of CMHs whose vascular expression was analyzed by qPCR.
