# Supplementary material for: Insulin‐like growth factor 1 deficiency exacerbates hypertension‐induced cerebral microhemorrhages in mice, mimicking the aging phenotype
Source: Aging Cell. 2017 Mar 14;16(3):469–79. doi: 10.1111/acel.12583 (PMC5418199; doi:10.1111/acel.12583)
Supplement: Supplementary file 1 — Table S1 List of important genes relevant for the structural integrity of the vasculature and potentially the pathogenesis of CMHs whose vascular expression was analyzed by qPCR. [file ACEL-16-469-s001.docx]

**Tarantini et al: “*IGF-1 deficiency exacerbates hypertension-induced cerebral microhemorrhages in mice, mimicking the aging phenotype”***

**Supplementary table 1:** List of important genes relevant for the structural integrity of the vasculature and potentially the pathogenesis of CMHs whose vascular expression was analyzed by qPCR. Genes whose up-regulation is expected to weaken the vascular wall were designated as “pro-fragility”, whereas genes whose down-regulation is expected to weaken the vascular wall were designated as “anti-fragility”. The genes included in this list were previously shown to be expressed in cerebral vessels at the mRNA (Csiszar and Ungvari 2016, unpublished data) and/or protein level([Badhwar *et al.* 2014](#_ENREF_5)). The role of these genes in cerebromicrovascular pathophysiology was predicted based on the results of clinical and/or experimental studies or inferred from their known action in regulation of the extracellular matrix and structural integrity of extracranial vessels. Arrows indicate the direction of gene expression changes observed in cerebral arteries of IGF-1 deficient mice, hypertensive control mice and hypertensive IGF-1 deficient mice.

| **Category** | **Symbol** | **Gene name** | **Description/function** | **Classification** | **Effect of IGF-1 deficiency** | **Change with hypertension (control)** | **Change with hypertension (IGF-1 deficient)** |
| --- | --- | --- | --- | --- | --- | --- | --- |
| *ECM components, ECM-associated proteins and factors involved in ECM remodeling* | *Col1a1* | collagen, type I, alpha 1 | - major component of type I collagen - provides structural support - type I collagen is expressed by smooth muscle cells ([Ponticos *et al.* 2004](#_ENREF_79)) - patients with COL1A1 mutation develop thoracic aorta aneurysm in 30% of the cases | Anti | → | → | → |
|  | *Col3a1* | collagen, type III, alpha 1 | - component of type III collagen - COL3A1 null mutations results in Vascular Ehlers-Danlos Syndrome (EDS type IV), which is characterized by arterial fragility | Anti | → | ↑ | → |
|  | *Col5a1* | collagen type V alpha 1 chain | - component of type V collagen - type V collagen molecules arrange themselves into long, thin fibrils with type I collagen. Type V collagen regulates the diameter of those fibrils. It also controls the assembly of other types of collagen into fibrils. - mutation of COL5A1 cause Ehlers-Danlos Syndrome, which also affects the vascular wall and cause arterial rupture([Borck *et al.* 2010](#_ENREF_10)) | Anti | → | → | ↓ |
|  | *Col5a2* | collagen type V alpha 2 chain | - component of type V collagen - type V collagen molecules arrange themselves into long, thin fibrils with type I collagen. Type V collagen regulates the diameter of those fibrils. It also controls the assembly of other types of collagen into fibrils. - mutation of COL5A2 has also been identified in Ehlers-Danlos Syndrome, and associated with aortic aneurysm and dissection([Meienberg *et al.* 2010](#_ENREF_64); [Ziganshin *et al.* 2015](#_ENREF_123)) | Anti | → | ↑ | → |
|  | *Col6a2* | collagen type VI alpha 2 chain | - component of type VI collagen - type VI collagen links basement membranes to nearby cells. - mutations in type VI collagen cause Ullrich’s disease, in which capillary abnormalities have been identified([Niiyama *et al.* 2003](#_ENREF_72)) | Anti | → | ↑ | → |
|  | *Col8a1* | collagen type VIII alpha 1 chain | - encodes one of the two alpha chains of type VIII collagen, which is a member of the short-chain nonfibrillar collagen family - type VIII collagen is necessary for migration and proliferation of vascular smooth muscle cells - upregulated after vascular injury; - protects fibrous cap from rupture | Anti | → | → | → |
|  | *Col12a1* | collagen type XII alpha 1 chain | - encodes the alpha chain of type XII collagen - type XII collagen interacts with type I collagen-containing fibrils - mutations in COL12A1 are associated with Bethlem myopathy([Hicks *et al.* 2014](#_ENREF_41)) - collagen  XII null mice demonstrate delayed endothelial maturation resulting in functional changes in endothelial function([Hemmavanh *et al.* 2013](#_ENREF_39)) - dysregulated in aortic aneurysm | Anti | → | → | → |
|  | *Col14a1* | collagen type XIV alpha 1 chain | - encodes the alpha chain of type XIV collagen - type XIV collagen interacts with the fibril surface and is involved in the regulation of fibrillogenesis - plays an adhesive role by integrating collagen bundles - collagen  XIV null mice demonstrate delayed endothelial maturation resulting in functional changes in endothelial function([Hemmavanh *et al.* 2013](#_ENREF_39)) | Anti | → | → | ↓ |
|  | *Col15a1* | collagen type XV alpha 1 chain | - encodes the alpha chain of type XV collagen - function to adhere basement membranes to underlying connective tissue stroma - COL15A1 null mice show impaired microvascular structure and endothelial cell degeneration([Eklund *et al.* 2001](#_ENREF_29); [Rasi *et al.* 2010](#_ENREF_82)) | Anti | → | → | → |
|  | *Col18a1* | collagen type XVIII alpha 1 chain | - encodes the alpha chain of type XVIII collagen - proteolytic processing at several endogenous cleavage sites in the C-terminal domain results in production of endostatin, a potent antiangiogenic protein - mutations in COL18A1 are associated with Knobloch syndrome, characterized by impaired microvascular structure ([Manon-Jensen *et al.* 2016](#_ENREF_62)) | Anti | → | ↑ | ↑ |
|  | *Col2a1* | collagen type II alpha 1 chain | - encodes the alpha-1 chain of type II collagen, a fibrillar collagen - expressed in blood vessels([Zhu *et al.* 2014](#_ENREF_122)) | Anti | → | → | → |
|  | *Col4a2* | collagen type IV alpha 2 chain | - encodes one of the six subunits of type IV collagen, the major structural component of basement membranes - mutations in COL4A2 associated with intracerebral hemorrhages([Gunda *et al.* 2014](#_ENREF_35)) and angiopathy (e.g. thickening of capillaries, aneurysms)([Plaisier *et al.* 2010](#_ENREF_77)) | Anti | ↓ | → | ↓ |
|  | *Eln* | elastin | - major component of elastic fibers - provides strength and flexibility to the arteries - mutations of *Eln* display vascular phenotypes ([Micale *et al.* 2010](#_ENREF_67); [Wint *et al.* 2014](#_ENREF_114)) - decreased elastin content associates with aneurysms([Carmo *et al.* 2002](#_ENREF_12)) - elastin gene hemideletion of Williams syndrome promotes stroke - elastase infusion causes cerebral aneurysms([Villano *et al.* 2012](#_ENREF_107)) | Anti | → | ↑ | ↑ |
|  | *Hspg2* | heparan sulfate proteoglycan 2 | - encodes the perlecan protein - key component of the vascular ECM - important for normal microvascular structure([Chang *et al.* 2015](#_ENREF_14)) - intracranial aneurysm susceptibility gene([Ruigrok *et al.* 2009](#_ENREF_86)) | Anti | → | → | → |
|  | *Vcan* | versican | - large ECM proteoglycan expressed in smooth muscle cells, which plays an important role in ECM assembly and the pathogenesis of intracranial aneurysms([Melrose *et al.* 1998](#_ENREF_65); [Ruigrok *et al.* 2006](#_ENREF_85); [Sathyan *et al.* 2014](#_ENREF_90)) - involved in regulation of cell adhesion, migration, and proliferation - versican interacts with a number of ECM components near the cell surface including hyaluronic acid, tenascin-R and -C, thrombospondin 1, fibronectin, and fibrillin - may modify cellular stiffness altering mechanotransduction | Anti | → | ↑ | ↑ |
|  | *Nid1* | entactin, nidogen 1 | - encodes a member of the nidogen family of basement membrane glycoproteins - role in cell interactions with the extracellular matrix - nidogen deficiency promotes hemorrhage([Bader *et al.* 2005](#_ENREF_4)) | Anti | → | → | → |
|  | *Fbn1* | fibrilin-1 | - required for assembly of the elastin fiber - mice lacking the FBN1 gene have thin, fragmented arterial elastic fibers leading to aortic aneurysms([Carta *et al.* 2006](#_ENREF_13)) - in humans, Fbn1 mutations lead to Marfan syndrome, which is characterized by vascular abnormalities ([Ramirez *et al.* 2007](#_ENREF_81)), including intracerebral hemorrhage([Neshige *et al.* 2014](#_ENREF_70)) - fibrillin-1 impairment in mice enhances blood-brain barrier permeability | Anti | → | ↑ | → |
|  | *Fbn2* | fibrilin-2 | - required for assembly of the elastin fiber - mutations in the *FBN2* gene have been found to cause congenital contractural arachnodactyly, which can manifest aortic dilatation and/or dissection([Takeda *et al.* 2015](#_ENREF_101)) | Anti | → | → | → |
|  | *Emilin1* | elastin microfibril interfacer 1 | - associates with elastic fibers at the interface between elastin and microfibrils - down-regulated in aneurysms([Zhang *et al.* 2015](#_ENREF_120)) - mice lacking the *Emilin1* gene exhibit hypertension and arteries with irregular elastic lamellae, smaller diameters, and thinner walls([Zanetti *et al.* 2004](#_ENREF_118); [Litteri *et al.* 2012](#_ENREF_58)) - target of MMP([Stegemann *et al.* 2013](#_ENREF_97)) | Anti | ↑ | → | → |
|  | *Emilin2* | elastin microfibril interfacer 2 | - responsible for anchoring smooth muscle cells to elastic fibers, and may be involved not only in the formation of the elastic fiber, but also in the processes that regulate vessel assembly | Anti | → | → | ↑ |
|  | *Fn1* | fibronectin 1 | - encodes for two types of the fibronectin-1 protein: soluble plasma fibronectin-1 and insoluble cellular fibronectin-1 - involved in blood clotting - impaired splicing of fibronectin may contribute to thoracic aortic aneurysm formation([Paloschi *et al.* 2011](#_ENREF_74)) | Anti | ↑ | → | → |
|  | *Fbln1* | fibulin-1 | - found in elastic tissues within the elastin core - mediates platelet adhesion via binding fibrinogen - mice deficient in Fbln1 present spontaneous bleeding defects associated with abnormal endothelial integrity in small blood vessels([Wagenseil & Mecham 2009](#_ENREF_108)) | Anti | → | → | → |
|  | *Fbln5* | fibulin-5 | - found in elastic tissues associated with microfibrils, critical for elastic fiber assembly - mice lacking Fbln5 have highly disrupted and disorganized elastic fibers, leading to defects in arterial blood vessels([Yanagisawa *et al.* 2002](#_ENREF_115)) - decreased in human aneurysms | Anti | → | ↑ | → |
|  | *Efemp2* | EGF containing fibulin like extracellular matrix protein 2 | - encodes for fibulin-4 - necessary for elastic fiber formation and connective tissue development - mutations in EFEMP2 result in cutis laxa type I (characterized by severe abnormalities involving the heart and blood vessels), ascending aortic aneurysms, arterial tortuosity along with other arterial defects([Papke & Yanagisawa 2014](#_ENREF_75)) and microvascular fragility([Hoyer *et al.* 2009](#_ENREF_44)) | Anti | → | → | → |
|  | *Sparc* | osteonectin | - ECM glycoprotein that plays a vital role in cell-matrix interactions, and collagen binding - Increases the production and activity of MMPs - SPARC is widely expressed in human intracranial aneurysms, and its expression correlates with MMP-2 and MMP-9 expression([Li *et al.* 2013a](#_ENREF_56)) | Pro | → | ↑ | ↑ |
|  | *Vtn* | vitronectin | - aneurysm wall prone to rupture contains decreased level of vitronectin - vitronectin-coated platinum coils showed efficacy occluding cerebral aneurysms([Whitlow *et al.* 2009](#_ENREF_112)) | Anti | → | → | → |
|  | *Bgn* | biglycan | - small leucine-rich repeat proteoglycan found in ECM - interacts with collagen - decreased expression in aneurysms([Theocharis & Karamanos 2002](#_ENREF_103); [Tang *et al.* 2014](#_ENREF_102)) - biglycan deficiency promotes aneurysms and aorta dissection([Heegaard *et al.* 2007](#_ENREF_38)) - BGN gene defects in humans cause an X-linked syndromic form of severe TAAD([Meester *et al.* 2016](#_ENREF_63)) | Anti | → | ↑ | → |
|  | *Dcn* | decorin | - small leucine-rich proteoglycan, which is similar to biglycan - binds to type I collagen fibrils, and plays a role in matrix assembly - Local administration of exogenous decorin prevents aneurysm development([Ueda *et al.* 2015](#_ENREF_106)) - down-regulated in aneurysms([Zhang *et al.* 2015](#_ENREF_120)) | Anti | → | ↑ | ↑ |
|  | *Thbs1* | thrombospondin 1 | - adhesive glycoprotein that mediates cell-to-cell and cell-to-matrix interactions - roles in platelet aggregation, angiogenesis, and tumorigenesis - loss-of-function of thrombospondin-1 impairs vascular homeostasis, promoting microvascular hemorrhage and sensitizing to abdominal aortic aneurysm ([Krishna *et al.* 2015](#_ENREF_54)) | Anti | → | ↑ | ↑ |
| *Proteases and their inhibitors* | *Mmp2* | matrix metallopeptidase 2 | - encodes matrix metallopeptidase 2 protein - one of the major function of MMP-2 is to cleave type IV collagen, which is the major component of basement membrane - thought to play an initiator role in abdominal aortic aneurysm([Thompson & Cockerill 2006](#_ENREF_104)) | Pro | → | ↑ | ↑ |
|  | *Mmp3* | matrix metallopeptidase 3 | - encodes matrix metallopeptidase 3 protein - MMP-3 protein degrades fibronectin, laminin, and collagens III, IV, IX and X - polymorphism of MMP-3 promotes aneurysm development([Saracini *et al.* 2012](#_ENREF_89)) | Pro | → | → | ↑ |
|  | *Mmp8* | matrix metallopeptidase 8 | - encodes matrix metallopeptidase 8 protein - involved in the degradation of type I, II and III collagen - a high concentration of MMP-8 is present in human abdominal aortic aneurysms([Wilson *et al.* 2005](#_ENREF_113)) and it correlates with increased risk for arterial disease([Pradhan-Palikhe *et al.* 2010](#_ENREF_80)) | Pro | → | → | ↑ |
|  | *Mmp9* | matrix metallopeptidase 9 | - encodes matrix metallopeptidase 9 protein - MMP-9 enzyme degrades type IV and V collagen - increased levels of MMP-9 contribute to human abdominal aortic aneurysms([Ciavarella *et al.* 2015](#_ENREF_17)) and its inhibition attenuates the formation of aneurysms in mouse models([Zhang *et al.* 2014](#_ENREF_121)) | Pro | ↑ | → | → |
|  | *Mmp12* | matrix metallopeptidase 12 | - encodes matrix metallopeptidase 12 protein - MMP-12 enzyme mostly degrades elastin but is also able to degrade a broad spectrum of ECM components([Didangelos *et al.* 2011](#_ENREF_25)) - increased activity of MMP-12 is associated with abdominal aortic aneurysm([Curci *et al.* 1998](#_ENREF_19); [Didangelos *et al.* 2011](#_ENREF_25)) and aortic dissection([Song *et al.* 2013](#_ENREF_95)) | Pro | ↑ | ↑ | ↑ |
|  | *Timp1* | tissue inhibitor of metalloproteinases 1 | - the proteins encoded by the TIMP gene family are natural inhibitors of the MMPs - protects against progression of cerebral aneurysms([Aoki *et al.* 2007](#_ENREF_2)) - lower levels of TIMPs in AD patients associate with microbleeds | Anti | → | ↑ | ↑ |
|  | *Timp2* | tissue inhibitor of metalloproteinases 2 | - protects against progression of cerebral aneurysms([Aoki *et al.* 2007](#_ENREF_2)) - TIMP-2 levels are lower in human abdominal aortic aneurysms and correlate with increased risk of arterial disease([Defawe *et al.* 2003](#_ENREF_24); [Pradhan-Palikhe *et al.* 2010](#_ENREF_80)) | Anti | → | ↑ | → |
|  | *Timp3* | tissue inhibitor of metalloproteinases 3 | - plays a critical role preserving arterial ECM in response to hypertension([Basu *et al.* 2013](#_ENREF_7)) | Anti | → | ↑ | ↑ |
|  | *Cma1* | chymase | - promotes ECM degradation - chymotrypsin-like serine protease([Doggrell & Wanstall 2004](#_ENREF_27)) - ACE-independent conversion of Ang I to Ang II - contributes to aneurysm formation. Chymase inhibitor prevents Ang II-induced aortic aneurysm development ([Inoue *et al.* 2009](#_ENREF_47)) | Pro | → | → | → |
| *Enzymes involved in ECM crosslinking* | *Lox* | lysyl oxidase | - initiates the crosslinking of collagens and elastin in the process of forming proper collagen fibers and elastic lamellae - defects in this gene have been linked with predisposition to thoracic aortic aneurysms and dissections([Lee *et al.* 2016](#_ENREF_55)) - decreased lysyl oxidase activity promotes rupture of internal elastic lamina([Osborne-Pellegrin *et al.* 1990](#_ENREF_73)) | Anti | → | ↑ | ↑ |
|  | *Loxl1* | lysyl oxidase-like 1 | - catalyzes the formation of lysine-derived crosslinks in collagen and elastin - guides elastin deposition in a spatially defined manner([Liu *et al.* 2004](#_ENREF_60)) - LOX/LOXL activity protects against aortic aneurysms in mice models([Remus *et al.* 2012](#_ENREF_83)) | Anti | → | ↑ | → |
|  | *Loxl2* | lysyl oxidase-like 2 | - catalyze the formation of lysine-derived crosslinks in collagen and elastin | Anti | → | ↑ | ↑ |
|  | *Loxl3* | lysyl oxidase-like 3 | - catalyze the formation of lysine-derived crosslinks in collagen and elastin | Anti | → | → | → |
|  | *Loxl4* | lysyl oxidase-like 4 | - catalyze the formation of lysine-derived crosslinks in collagen and elastin | Anti | → | ↑ | ↑ |
|  | *Plod1* | procollagen-lysine, 2-oxoglutarate 5-dioxygenase 1 | - lysyl hydroxylase is a membrane-bound protein localized to the endoplasmic reticulum. The enzyme catalyzes the hydroxylation of lysyl residues in collagen-like peptides. Hydroxylysyl groups are attachment sites for carbohydrates in collagen and thus are critical for the stability of intermolecular crosslinks. Some patients with Ehlers-Danlos syndrome type VI have deficiencies in lysyl hydroxylase activity ([Yeowell & Walker 2000](#_ENREF_116); [Walker *et al.* 2005](#_ENREF_109)). These patients demonstrate arterial rupture - about 15% of Plod1 knockout die of aortic rupture([Takaluoma *et al.* 2007](#_ENREF_99)) | Anti | ↑ | ↑ | → |
|  | *Tgm2* | transglutaminase 2 | - transglutaminases introduce cross-links between protein chains and have been implicated in arterial repair and prevention of aneurysm development ([Munezane *et al.* 2010](#_ENREF_68)) | Anti | → | ↑ | → |
| *Factors relevant for the structural integrity of the vascular wall and/or involved in the pathogenesis of hemorrhages* | *Acta2* | actin, alpha 2, smooth muscle | - most abundant protein in adult vascular smooth muscle cells - confers the force-generating capacity of the vascular smooth muscle cells - regulates vascular contractility([Schildmeyer *et al.* 2000](#_ENREF_93)) - mutations in this gene are associated with increased risk of thoracic aortic aneurysms([Disabella *et al.* 2011](#_ENREF_26)) (familial thoracic aortic aneurysm and dissection) | Anti | → | ↑ | → |
|  | *Vim* | vimentin | - member of the intermediate filament family - responsible for maintaining cell shape, integrity of the cytoplasm, and stabilizing cytoskeletal interactions - plays an important role in vascular development([Boraas & Ahsan 2016](#_ENREF_9)) - knock-out mice have exhibit impaired vascular structure ([Henrion *et al.* 1997](#_ENREF_40); [Schiffers *et al.* 2000](#_ENREF_92); [Liu *et al.* 2014](#_ENREF_59)) | Anti | → | → | → |
|  | *Des* | desmin | - desmin is a type III intermediate filament in smooth muscle cells, which regulates cytoarchitecture - SMCs in both non-ruptured and ruptured aneurysmal walls show no staining for desmin([Nakajima *et al.* 2000](#_ENREF_69)) | Anti | → | → | ↑ |
|  | *Plat* | plasminogen activator, tissue type | - catalyzes the conversion of plasminogen to plasmin, the major enzyme responsible for clot breakdown - increased enzymatic activity causes hyperfibrinolysis, which manifests as excessive bleeding. Treatment of stroke patients with recombinant tissue plasminogen activator increases the risk for intracerebral hemorrhage - by controlling plasmin-mediated proteolysis, it plays an important role in tissue remodeling and degradation - vascular expression is up-regulated in hypertension - promotes ECM degradation within vessel walls([Jiang *et al.* 2003](#_ENREF_48); [Hao *et al.* 2005](#_ENREF_36)) | Pro | → | ↑ | ↑ |
|  | *Tjp1* | tight junction protein 1 | - encodes a protein located on the cytoplasmic membrane surface of intercellular tight junctions. - may be involved in assembly, stabilization of tight junctions - highly expressed in endothelial cells - endothelial tight junctions are the most important structural component of the blood brain barrier, and their alteration can disrupt the control of vascular permeability([Nico & Ribatti 2012](#_ENREF_71)) | Anti | ↑ | → | → |
|  | *Cldn5* | claudin 5 | - claudins are integral membrane proteins and components of tight junctions - reduced expression of claudin-5 plays a crucial role in increased vascular permeability([Kitajima *et al.* 2006](#_ENREF_51)) - downregulation of claudin-5 expression has been linked to hemorrhages([D'Agnillo *et al.* 2013](#_ENREF_20)) | Anti | → | → | → |
|  | *Ocln* | occludin | - encodes an integral membrane protein that plays a role in the formation and regulation of the tight junctions - displacement of occludin is associated with blood brain barrier disruption in a model of stroke and spontaneous cerebral hemorrhage([Zinnanti *et al.* 2014](#_ENREF_124)) - cerebral amyloid angiopathy patients, who suffer spontaneous hemorrhages, show occluding deficiency in cerebral vessels([Cheng *et al.* 2014](#_ENREF_15)) | Anti | ↑ | → | ↓ |
| *Factors involved in vascular redox regulation and ROS-mediated MMP activation* | *Cybb* | cytochrome b-245 beta chain | - subunit of NADPH oxidase (NOX2) - in the arterial wall NOX is the major sources of ROS ([Drummond *et al.* 2011](#_ENREF_28)), which promote MMP activation and ECM degradation - up-regulation/activation of NOX is causally linked to the pathogenesis of cerebral microhemorrhages([Toth *et al.* 2015](#_ENREF_105)) - NOX2 overexpression increases the susceptibility of the aortic wall to Ang II-mediated aortic dissection([Fan *et al.* 2014](#_ENREF_30)) | Pro | → | ↑ | ↑ |
|  | *Nox4* | NADPH oxidase 4 | - member of the NOX family of NADPH oxidases - Nox4-/- mice are protected from angiotensin-induced cerebral microhemorrhages (Tarantini and Ungvari, unpublished data) | Pro | → | ↑ | ↑ |
|  | *Ncf1* | neutrophil cytosol factor 1 | - 47 kDa cytosolic subunit of neutrophil NADPH oxidase (p47phox), which is vital for NOX activation | Pro | ↑ | ↑ | ↑ |
|  | *Ncf2* | neutrophil cytosol factor 2 | - 67 kDa cytosolic subunit of neutrophil NADPH oxidase (p67phox), which is vital for NOX activation | Pro | → | → | → |
|  | *Nfe2l2* | nuclear factor, erythroid 2 like 2 (Nrf2) | - encodes the transcription factor Nrf2 that regulates genes which contain antioxidant response elements (ARE) in their promoters - important for the coordinated adaptive up-regulation of cytoprotective genes in response to oxidative stress - pharmacological activation of Nrf2 confers protection against cerebral microhemorrhages in a mouse model([Toth *et al.* 2015](#_ENREF_105)) | Anti | → | → | ↓ |
| *Factors that regulate vascular remodeling processes* | *Tgfb1* | transforming growth factor, beta 1 | - TGFß-1 protein helps to control cell proliferation, motility, differentiation and apoptosis. Also plays a role in blood vessel formation - dysregulated TGFβ signaling is involved in the pathogenesis of aortic aneurysmal disease([Holm *et al.* 2011](#_ENREF_42); [Gillis *et al.* 2013](#_ENREF_33)) - Loeys-Dietz syndrome (LDS) is a connective tissue disorder that is characterized by a high risk for aneurysm and dissection throughout the arterial tree and phenotypically resembles Marfan syndrome. Mutations in either TGF-β receptor gene (TGFBR1 or TGFBR2), which are predicted to result in diminished TGF-β signaling, cause LDS, which associate with widespread aortic and arterial aneurysms - knockin mouse strains with LDS mutations and a transgenic mouse overexpressing mutant Tgfbr2 recapitulate the LDS phenotype. However, these models seem to exhibit paradoxical compensatory increases in TGF-β signaling([Gallo *et al.* 2014](#_ENREF_32)). - During development loss of TGFβ1-TGFBR2-ALK5-Smad3 signaling promotes cerebral hemorrhages([Arnold *et al.* 2014](#_ENREF_3)). | ?Anti | → | ↑ | ↑ |
|  | *Tgfb2* | transforming growth factor, beta 2 | - regulates proliferation, differentiation, adhesion, migration - dysregulated TGFβ signaling is involved in the pathogenesis of aortic aneurysmal disease([Holm *et al.* 2011](#_ENREF_42); [Gillis *et al.* 2013](#_ENREF_33)) - mutation causes LDS type 4, which associate with aorta aneurysm - increased susceptibility to hypertension-induced CMHs in aged mice is associated with dysregulation of *Tgfb2* expression in cerebral vessels (unpublished observation) | ?Anti | → | → | → |
|  | *Tgfb3* | transforming growth factor, beta 3 | - mutation of the gene can lead to Loeys-Dietz syndrome 5, which is characterized by arterial tortuosity and aneurysms - multiple TGFB3 mutations cause syndromic presentations of aortic aneurysms([Bertoli-Avella *et al.* 2015](#_ENREF_8)) - increased susceptibility to hypertension-induced CMHs in aged mice is associated with dysregulation of *Tgfb3* expression in cerebral vessels (unpublished observation) | ?Anti | → | → | ↓ |
|  | *Spp1* | secreted phosphoprotein 1, osteopontin | - cytokine; marker of the vascular smooth muscle synthetic phenotype - osteopontin was proposed to promote aneurysm pathogenesis([Spin *et al.* 2011](#_ENREF_96); [Yuan *et al.* 2011](#_ENREF_117); [Huusko *et al.* 2013](#_ENREF_45); [Meng *et al.* 2014](#_ENREF_66)) | Pro | → | ↑ | ↑ |
|  | *Tnr* | tenascin R | - member of the tenascin family of ECM glycoproteins secreted by smooth muscle cells - tenascins play a role in hypertension induced vascular remodeling - tenascin family members are upregulated in aneurysms ([Satoh *et al.* 2010](#_ENREF_91)), protecting the vascular wall from rupture([Imanaka-Yoshida & Aoki 2014](#_ENREF_46); [Kimura *et al.* 2014](#_ENREF_50)) | Anti | → | → | → |
|  | *Pgf* | Placental growth factor | - PGF is a member of the VEGF sub-family - mechanosenitive gene expression: aortic expression changes in hypertension - promotes ECM remodeling, healing of tissue damage - Pgf(-/-) mice rapidly die of heart failure within 1 week of pressure overload, their vasculature show an inability to adapt to hypertension([Accornero *et al.* 2011](#_ENREF_1)) | Anti | → | → | → |
|  | *Fgf2* | fibroblast growth factor 2 | - present in basement membranes and in the subendothelial extracellular matrix of blood vessels - involved in the regulation of cell survival, cell division, and angiogenesis - FGFs maintain and promote vascular integrity; - FGFs protect the blood-brain barrier - its controlled release has been widely proven to repair artery aneurysms([Hoshina *et al.* 2004](#_ENREF_43); [Jiao *et al.* 2012](#_ENREF_49)) | Anti | → | ↑ | ↑ |
|  | *Igf1* | inulin-like growth factor 1 | - in addition to circulating IGF-1 there is also a paracrine IGF-1 system in the vascular wall, which may or may not compensate for circulating IGF-1 deficiency | Anti | → | → | → |
|  | *Ctgf* | connective tissue growth factor, CCN2 | - matricellular protein of the CCN family of ECM-associated heparin-binding proteins, which regulates cell adhesion, migration, proliferation by binding to various cell surface proteins including integrin receptors and cell surface heparan sulfate proteoglycans([Ponticos 2013](#_ENREF_78)) - binds growth factor ligands such as BMP4, TGFβ, and VEGF thereby regulating their receptor binding affinity - increased vascular wall stress is thought to promote changes in VSMC towards a synthetic phenotype, which is mediated by ROS accumulation via CTGF([Branchetti *et al.* 2013](#_ENREF_11); [Meng *et al.* 2014](#_ENREF_66)) - increased in aneurysms([Wang *et al.* 2006](#_ENREF_111)) - down-regulation in aneurysmal disease may be protective([Zhan *et al.* 2015](#_ENREF_119)) - up-regulated by TGFβ - up-regulated in angiotensin II induced hypertension ([Ruperez *et al.* 2003a](#_ENREF_87); [Ruperez *et al.* 2003b](#_ENREF_88); [de las Heras *et al.* 2006](#_ENREF_23); [de las Heras *et al.* 2007](#_ENREF_22)) - contributes to microvascular remodeling in hypertensive patients via an angiotensin II dependent manner([Gomez-Garre *et al.* 2006](#_ENREF_34)) - may stablize atherosclerotic plaques - in diabetic retinopathy CTGF contributes to thickening of the retinal capillary basal lamina and is involved in loss of pericytes([Klaassen *et al.* 2015](#_ENREF_52)). | ?Anti | → | ↑ | ↑ |
|  | *Edn1* | endothelin 1 | - regulates vascular remodeling([Dao *et al.* 2006](#_ENREF_21)) - endothelin 1 overexpression can induce aneurysms in mouse models([Li *et al.* 2013b](#_ENREF_57)) | Pro | → | → | → |
|  | *Il6* | interleukin-6 | - pro-inflammatory cytokine - IL-6 signaling is causally involved in aneurysm pathogenesis([Harrison *et al.* 2013](#_ENREF_37)) - increased levels associate with aneurysm rupture([Cheuk & Cheng 2007](#_ENREF_16)) | Pro | → | ↑ | → |
|  | *Mfge8* | milk fat globule-egf factor 8 protein | - encodes a preprotein, that is proteolytically processed to multiple protein products - the major product, lactadherin, is implicated in wound healing and promotes VEGF-dependent neovascularization. Lactadherin can be further processed to medin, which comprises the major protein component of aortic medial amyloid - the upregulation of MFGE8 and its derived medin are closely associated with adverse arterial remodelling in aging, hypertension, aneurysm and dissection([Wang *et al.* 2013](#_ENREF_110)) | Pro | → | → | → |
|  | *Ppbp* | Cxcl7,  Pro-Platelet basic protein,  NAP-2 (neutrophil activating peptide 2) | - stimulates synthesis of extracellular matrix - exerts procoagulant effects([Kowalska *et al.* 2010](#_ENREF_53)) - expressed in blood vessels and cultured smooth muscle cells, in addition to its known expression in platelets - expressed in atherosclerotic plaques([Perisic *et al.* 2016](#_ENREF_76)) - isoform of β-thromboglobulin | ?Anti | ↓ | ↓ | ↓ |
|  | *Hpse* | Heparanase | - heparan sulfate proteoglycans found in the ECM (e.g. perlecan, agrin and collagen XVIII) are potent regulators of vascular remodeling and repair - heparanase is the major enzyme capable of degrading heparan sulfate in mammalian cells - heparanase plays a role in the regulation of arterial structure, mechanics, and repair([Baker *et al.* 2009](#_ENREF_6)) - transgenic mice overexpressing heparanase had increased arterial thickness, cellular density, and mechanical compliance | Anti | → | → | → |
|  | *Adam17* | ADAM metallopeptidase domain 17 | - encodes a member of ADAM family - members of this family are involved in cell-cell and cell-matrix interactions - ADAM 17 inhibitory antibody prevented ANG II infusion induced vascular remodeling in mice([Takayanagi *et al.* 2016](#_ENREF_100)) - it has been implicated in the loss of microvascular barrier function under hypoxia([Cui *et al.* 2015](#_ENREF_18)) and has been identified in human and mouse aortic aneurysms([Spin *et al.* 2011](#_ENREF_96); [Folkesson *et al.* 2015](#_ENREF_31)) | Pro | → | → | → |
|  | *Adam10* | ADAM metallopeptidase domain 10 | - encodes a member of ADAM family - cleaves many proteins including TNF-alpha and E-cadherin - increased ADAM10 expression is associated with an increase in endothelial permeability([Schulz *et al.* 2008](#_ENREF_94)) and has been identified in human abdominal aortic aneurysms([Folkesson *et al.* 2015](#_ENREF_31)) | Pro | → | → | → |
|  | *Adam15* | ADAM metallopeptidase domain 15 | - encodes a member of ADAM family - active metalloproteinase with gelatinolytic and collagenolytic activity - mediator of mechanisms underlying inflammatory diseases - ADAM15 induces hyperpermeability of the endothelial barrier in vitro([Sun *et al.* 2010](#_ENREF_98)) | Pro | → | → | → |
|  | *Adamts1* | ADAM metallopeptidase with thrombospondin type 1 motif 1 | - encodes a member of the ADAMTS protein family - ADAMTS is a family of multidomain extracellular protease enzymes - ADAMTS family is involved in ECM degradation([Lu *et al.* 2011](#_ENREF_61)) - ADAMTS-1 levels are elevated in thoracic aortic aneurysms and dissections([Ren *et al.* 2013](#_ENREF_84)) | Pro | ↑ | ↑ | → |

**References**

Accornero F, van Berlo JH, Benard MJ, Lorenz JN, Carmeliet P , Molkentin JD (2011). Placental growth factor regulates cardiac adaptation and hypertrophy through a paracrine mechanism. *Circ Res*. **109**, 272-280.

Aoki T, Kataoka H, Moriwaki T, Nozaki K , Hashimoto N (2007). Role of TIMP-1 and TIMP-2 in the progression of cerebral aneurysms. *Stroke*. **38**, 2337-2345.

Arnold TD, Niaudet C, Pang MF, Siegenthaler J, Gaengel K, Jung B, Ferrero GM, Mukouyama YS, Fuxe J, Akhurst R, Betsholtz C, Sheppard D , Reichardt LF (2014). Excessive vascular sprouting underlies cerebral hemorrhage in mice lacking alphaVbeta8-TGFbeta signaling in the brain. *Development*. **141**, 4489-4499.

Bader BL, Smyth N, Nedbal S, Miosge N, Baranowsky A, Mokkapati S, Murshed M , Nischt R (2005). Compound genetic ablation of nidogen 1 and 2 causes basement membrane defects and perinatal lethality in mice. *Mol Cell Biol*. **25**, 6846-6856.

Badhwar A, Stanimirovic DB, Hamel E , Haqqani AS (2014). The proteome of mouse cerebral arteries. *J Cereb Blood Flow Metab*. **34**, 1033-1046.

Baker AB, Groothuis A, Jonas M, Ettenson DS, Shazly T, Zcharia E, Vlodavsky I, Seifert P , Edelman ER (2009). Heparanase alters arterial structure, mechanics, and repair following endovascular stenting in mice. *Circ Res*. **104**, 380-387.

Basu R, Lee J, Morton JS, Takawale A, Fan D, Kandalam V, Wang X, Davidge ST , Kassiri Z (2013). TIMP3 is the primary TIMP to regulate agonist-induced vascular remodelling and hypertension. *Cardiovasc Res*. **98**, 360-371.

Bertoli-Avella AM, Gillis E, Morisaki H, Verhagen JM, de Graaf BM, van de Beek G, Gallo E, Kruithof BP, Venselaar H, Myers LA, Laga S, Doyle AJ, Oswald G, van Cappellen GW, Yamanaka I, van der Helm RM, Beverloo B, de Klein A, Pardo L, Lammens M, Evers C, Devriendt K, Dumoulein M, Timmermans J, Bruggenwirth HT, Verheijen F, Rodrigus I, Baynam G, Kempers M, Saenen J, Van Craenenbroeck EM, Minatoya K, Matsukawa R, Tsukube T, Kubo N, Hofstra R, Goumans MJ, Bekkers JA, Roos-Hesselink JW, van de Laar IM, Dietz HC, Van Laer L, Morisaki T, Wessels MW , Loeys BL (2015). Mutations in a TGF-beta ligand, TGFB3, cause syndromic aortic aneurysms and dissections. *J Am Coll Cardiol*. **65**, 1324-1336.

Boraas LC , Ahsan T (2016). Lack of vimentin impairs endothelial differentiation of embryonic stem cells. *Sci Rep*. **6**, 30814.

Borck G, Beighton P, Wilhelm C, Kohlhase J , Kubisch C (2010). Arterial rupture in classic Ehlers-Danlos syndrome with COL5A1 mutation. *Am J Med Genet A*. **152A**, 2090-2093.

Branchetti E, Poggio P, Sainger R, Shang E, Grau JB, Jackson BM, Lai EK, Parmacek MS, Gorman RC, Gorman JH, Bavaria JE , Ferrari G (2013). Oxidative stress modulates vascular smooth muscle cell phenotype via CTGF in thoracic aortic aneurysm. *Cardiovasc Res*. **100**, 316-324.

Carmo M, Colombo L, Bruno A, Corsi FR, Roncoroni L, Cuttin MS, Radice F, Mussini E , Settembrini PG (2002). Alteration of elastin, collagen and their cross-links in abdominal aortic aneurysms. *Eur J Vasc Endovasc Surg*. **23**, 543-549.

Carta L, Pereira L, Arteaga-Solis E, Lee-Arteaga SY, Lenart B, Starcher B, Merkel CA, Sukoyan M, Kerkis A, Hazeki N, Keene DR, Sakai LY , Ramirez F (2006). Fibrillins 1 and 2 perform partially overlapping functions during aortic development. *J Biol Chem*. **281**, 8016-8023.

Chang YT, Tseng CN, Tannenberg P, Eriksson L, Yuan K, de Jesus Perez VA, Lundberg J, Lengquist M, Botusan IR, Catrina SB, Tran PK, Hedin U , Tran-Lundmark K (2015). Perlecan heparan sulfate deficiency impairs pulmonary vascular development and attenuates hypoxic pulmonary hypertension. *Cardiovasc Res*. **107**, 20-31.

Cheng X, He P, Yao H, Dong Q, Li R , Shen Y (2014). Occludin deficiency with BACE1 elevation in cerebral amyloid angiopathy. *Neurology*. **82**, 1707-1715.

Cheuk BL , Cheng SW (2007). Differential secretion of prostaglandin E(2), thromboxane A(2) and interleukin-6 in intact and ruptured abdominal aortic aneurysms. *Int J Mol Med*. **20**, 391-395.

Ciavarella C, Alviano F, Gallitto E, Ricci F, Buzzi M, Velati C, Stella A, Freyrie A , Pasquinelli G (2015). Human Vascular Wall Mesenchymal Stromal Cells Contribute to Abdominal Aortic Aneurysm Pathogenesis Through an Impaired Immunomodulatory Activity and Increased Levels of Matrix Metalloproteinase-9. *Circ J*. **79**, 1460-1469.

Cui D, Arima M, Takubo K, Kimura T, Horiuchi K, Minagawa T, Matsuda S , Ikeda E (2015). ADAM12 and ADAM17 are essential molecules for hypoxia-induced impairment of neural vascular barrier function. *Sci Rep*. **5**, 12796.

Curci JA, Liao S, Huffman MD, Shapiro SD , Thompson RW (1998). Expression and localization of macrophage elastase (matrix metalloproteinase-12) in abdominal aortic aneurysms. *J Clin Invest*. **102**, 1900-1910.

D'Agnillo F, Williams MC, Moayeri M , Warfel JM (2013). Anthrax lethal toxin downregulates claudin-5 expression in human endothelial tight junctions. *PLoS One*. **8**, e62576.

Dao HH, Bouvet C, Moreau S, Beaucage P, Larivière R, Servant MJ, de Champlain J , Moreau P (2006). Endothelin is a dose-dependent trophic factor and a mitogen in small arteries in vivo. *Cardiovasc Res*. **71**, 61-68.

de las Heras N, Ruiz-Ortega M, Miana M, Ruperez M, Sanz-Rosa D, Aragoncillo P, Mezzano S, Cachofeiro V, Egido J , Lahera V (2007). Interactions between aldosterone and connective tissue growth factor in vascular and renal damage in spontaneously hypertensive rats. *J Hypertens*. **25**, 629-638.

de las Heras N, Ruiz-Ortega M, Ruperez M, Sanz-Rosa D, Miana M, Aragoncillo P, Mezzano S, Lahera V, Egido J , Cachofeiro V (2006). Role of connective tissue growth factor in vascular and renal damage associated with hypertension in rats. Interactions with angiotensin II. *J Renin Angiotensin Aldosterone Syst*. **7**, 192-200.

Defawe OD, Colige A, Lambert CA, Munaut C, Delvenne P, Lapière CM, Limet R, Nusgens BV , Sakalihasan N (2003). TIMP-2 and PAI-1 mRNA levels are lower in aneurysmal as compared to athero-occlusive abdominal aortas. *Cardiovasc Res*. **60**, 205-213.

Didangelos A, Yin X, Mandal K, Saje A, Smith A, Xu Q, Jahangiri M , Mayr M (2011). Extracellular matrix composition and remodeling in human abdominal aortic aneurysms: a proteomics approach. *Mol Cell Proteomics*. **10**, M111 008128.

Disabella E, Grasso M, Gambarin FI, Narula N, Dore R, Favalli V, Serio A, Antoniazzi E, Mosconi M, Pasotti M, Odero A , Arbustini E (2011). Risk of dissection in thoracic aneurysms associated with mutations of smooth muscle alpha-actin 2 (ACTA2). *Heart*. **97**, 321-326.

Doggrell SA , Wanstall JC (2004). Vascular chymase: pathophysiological role and therapeutic potential of inhibition. *Cardiovasc Res*. **61**, 653-662.

Drummond GR, Selemidis S, Griendling KK , Sobey CG (2011). Combating oxidative stress in vascular disease: NADPH oxidases as therapeutic targets. *Nat Rev Drug Discov*. **10**, 453-471.

Eklund L, Piuhola J, Komulainen J, Sormunen R, Ongvarrasopone C, Fássler R, Muona A, Ilves M, Ruskoaho H, Takala TE , Pihlajaniemi T (2001). Lack of type XV collagen causes a skeletal myopathy and cardiovascular defects in mice. *Proc Natl Acad Sci U S A*. **98**, 1194-1199.

Fan LM, Douglas G, Bendall JK, McNeill E, Crabtree MJ, Hale AB, Mai A, Li JM, McAteer MA, Schneider JE, Choudhury RP , Channon KM (2014). Endothelial cell-specific reactive oxygen species production increases susceptibility to aortic dissection. *Circulation*. **129**, 2661-2672.

Folkesson M, Li C, Frebelius S, Swedenborg J, Wågsäter D, Williams KJ, Eriksson P, Roy J , Liu ML (2015). Proteolytically active ADAM10 and ADAM17 carried on membrane microvesicles in human abdominal aortic aneurysms. *Thromb Haemost*. **114**, 1165-1174.

Gallo EM, Loch DC, Habashi JP, Calderon JF, Chen Y, Bedja D, van Erp C, Gerber EE, Parker SJ, Sauls K, Judge DP, Cooke SK, Lindsay ME, Rouf R, Myers L, ap Rhys CM, Kent KC, Norris RA, Huso DL , Dietz HC (2014). Angiotensin II-dependent TGF-beta signaling contributes to Loeys-Dietz syndrome vascular pathogenesis. *J Clin Invest*. **124**, 448-460.

Gillis E, Van Laer L , Loeys BL (2013). Genetics of thoracic aortic aneurysm: at the crossroad of transforming growth factor-β signaling and vascular smooth muscle cell contractility. *Circ Res*. **113**, 327-340.

Gomez-Garre D, Martin-Ventura JL, Granados R, Sancho T, Torres R, Ruano M, Garcia-Puig J , Egido J (2006). Losartan improves resistance artery lesions and prevents CTGF and TGF-beta production in mild hypertensive patients. *Kidney Int*. **69**, 1237-1244.

Gunda B, Mine M, Kovács T, Hornyák C, Bereczki D, Várallyay G, Rudas G, Audrezet MP , Tournier-Lasserve E (2014). COL4A2 mutation causing adult onset recurrent intracerebral hemorrhage and leukoencephalopathy. *J Neurol*. **261**, 500-503.

Hao Z, Jiang X, Sharafeih R, Shen S, Hand AR, Cone RE , O'Rourke J (2005). Stimulated release of tissue plasminogen activator from artery wall sympathetic nerves: implications for stress-associated wall damage. *Stress*. **8**, 141-149.

Harrison SC, Smith AJ, Jones GT, Swerdlow DI, Rampuri R, Bown MJ, Folkersen L, Baas AF, de Borst GJ, Blankensteijn JD, Price JF, van der Graaf Y, McLachlan S, Agu O, Hofman A, Uitterlinden AG, Franco-Cereceda A, Ruigrok YM, van't Hof FN, Powell JT, van Rij AM, Casas JP, Eriksson P, Holmes MV, Asselbergs FW, Hingorani AD , Humphries SE (2013). Interleukin-6 receptor pathways in abdominal aortic aneurysm. *Eur Heart J*. **34**, 3707-3716.

Heegaard AM, Corsi A, Danielsen CC, Nielsen KL, Jorgensen HL, Riminucci M, Young MF , Bianco P (2007). Biglycan deficiency causes spontaneous aortic dissection and rupture in mice. *Circulation*. **115**, 2731-2738.

Hemmavanh C, Koch M, Birk DE , Espana EM (2013). Abnormal corneal endothelial maturation in collagen XII and XIV null mice. *Invest Ophthalmol Vis Sci*. **54**, 3297-3308.

Henrion D, Terzi F, Matrougui K, Duriez M, Boulanger CM, Colucci-Guyon E, Babinet C, Briand P, Friedlander G, Poitevin P , Lévy BI (1997). Impaired flow-induced dilation in mesenteric resistance arteries from mice lacking vimentin. *J Clin Invest*. **100**, 2909-2914.

Hicks D, Farsani GT, Laval S, Collins J, Sarkozy A, Martoni E, Shah A, Zou Y, Koch M, Bönnemann CG, Roberts M, Lochmüller H, Bushby K , Straub V (2014). Mutations in the collagen XII gene define a new form of extracellular matrix-related myopathy. *Hum Mol Genet*. **23**, 2353-2363.

Holm TM, Habashi JP, Doyle JJ, Bedja D, Chen Y, van Erp C, Lindsay ME, Kim D, Schoenhoff F, Cohn RD, Loeys BL, Thomas CJ, Patnaik S, Marugan JJ, Judge DP , Dietz HC (2011). Noncanonical TGFβ signaling contributes to aortic aneurysm progression in Marfan syndrome mice. *Science*. **332**, 358-361.

Hoshina K, Koyama H, Miyata T, Shigematsu H, Takato T, Dalman RL , Nagawa H (2004). Aortic wall cell proliferation via basic fibroblast growth factor gene transfer limits progression of experimental abdominal aortic aneurysm. *J Vasc Surg*. **40**, 512-518.

Hoyer J, Kraus C, Hammersen G, Geppert JP , Rauch A (2009). Lethal cutis laxa with contractural arachnodactyly, overgrowth and soft tissue bleeding due to a novel homozygous fibulin-4 gene mutation. *Clin Genet*. **76**, 276-281.

Huusko T, Salonurmi T, Taskinen P, Liinamaa J, Juvonen T, Pääkkö P, Savolainen M , Kakko S (2013). Elevated messenger RNA expression and plasma protein levels of osteopontin and matrix metalloproteinase types 2 and 9 in patients with ascending aortic aneurysms. *J Thorac Cardiovasc Surg*. **145**, 1117-1123.

Imanaka-Yoshida K , Aoki H (2014). Tenascin-C and mechanotransduction in the development and diseases of cardiovascular system. *Front Physiol*. **5**, 283.

Inoue N, Muramatsu M, Jin D, Takai S, Hayashi T, Katayama H, Kitaura Y, Tamai H , Miyazaki M (2009). Effects of chymase inhibitor on angiotensin II-induced abdominal aortic aneurysm development in apolipoprotein E-deficient mice. *Atherosclerosis*. **204**, 359-364.

Jiang X, Hand AR, Shen S, Cone RE , O'Rourke J (2003). Enhanced tissue plasminogen activator synthesis by the sympathetic neurons that innervate aging vessels. *J Neurosci Res*. **71**, 567-574.

Jiao L, Jiang M, Fang J, Deng Y, Chen Z , Wu M (2012). Basic fibroblast growth factor gene transfection in repair of internal carotid artery aneurysm wall. *Neural Regen Res*. **7**, 2915-2921.

Kimura T, Shiraishi K, Furusho A, Ito S, Hirakata S, Nishida N, Yoshimura K, Imanaka-Yoshida K, Yoshida T, Ikeda Y, Miyamoto T, Ueno T, Hamano K, Hiroe M, Aonuma K, Matsuzaki M, Imaizumi T , Aoki H (2014). Tenascin C protects aorta from acute dissection in mice. *Sci Rep*. **4**, 4051.

Kitajima Y, Endo T, Nagasawa K, Manase K, Honnma H, Baba T, Hayashi T, Chiba H, Sawada N , Saito T (2006). Hyperstimulation and a gonadotropin-releasing hormone agonist modulate ovarian vascular permeability by altering expression of the tight junction protein claudin-5. *Endocrinology*. **147**, 694-699.

Klaassen I, van Geest RJ, Kuiper EJ, van Noorden CJ , Schlingemann RO (2015). The role of CTGF in diabetic retinopathy. *Exp Eye Res*. **133**, 37-48.

Kowalska MA, Rauova L , Poncz M (2010). Role of the platelet chemokine platelet factor 4 (PF4) in hemostasis and thrombosis. *Thromb Res*. **125**, 292-296.

Krishna SM, Seto SW, Jose RJ, Biros E, Moran CS, Wang Y, Clancy P , Golledge J (2015). A peptide antagonist of thrombospondin-1 promotes abdominal aortic aneurysm progression in the angiotensin II-infused apolipoprotein-E-deficient mouse. *Arterioscler Thromb Vasc Biol*. **35**, 389-398.

Lee VS, Halabi CM, Hoffman EP, Carmichael N, Leshchiner I, Lian CG, Bierhals AJ, Vuzman D, Mecham RP, Frank NY, Stitziel NO , Medicine BG (2016). Loss of function mutation in LOX causes thoracic aortic aneurysm and dissection in humans. *Proc Natl Acad Sci U S A*. **113**, 8759-8764.

Li B, Li F, Chi L, Zhang L , Zhu S (2013a). The expression of SPARC in human intracranial aneurysms and its relationship with MMP-2/-9. *PLoS One*. **8**, e58490.

Li MW, Mian MO, Barhoumi T, Rehman A, Mann K, Paradis P , Schiffrin EL (2013b). Endothelin-1 overexpression exacerbates atherosclerosis and induces aortic aneurysms in apolipoprotein E knockout mice. *Arterioscler Thromb Vasc Biol*. **33**, 2306-2315.

Litteri G, Carnevale D, D'Urso A, Cifelli G, Braghetta P, Damato A, Bizzotto D, Landolfi A, Ros FD, Sabatelli P, Facchinello N, Maffei A, Volpin D, Colombatti A, Bressan GM , Lembo G (2012). Vascular smooth muscle Emilin-1 is a regulator of arteriolar myogenic response and blood pressure. *Arterioscler Thromb Vasc Biol*. **32**, 2178-2184.

Liu T, Ghamloush MM, Aldawood A, Warburton R, Toksoz D, Hill NS, Tang DD , Kayyali US (2014). Modulating endothelial barrier function by targeting vimentin phosphorylation. *J Cell Physiol*. **229**, 1484-1493.

Liu X, Zhao Y, Gao J, Pawlyk B, Starcher B, Spencer JA, Yanagisawa H, Zuo J , Li T (2004). Elastic fiber homeostasis requires lysyl oxidase-like 1 protein. *Nat Genet*. **36**, 178-182.

Lu P, Takai K, Weaver VM , Werb Z (2011). Extracellular matrix degradation and remodeling in development and disease. *Cold Spring Harb Perspect Biol*. **3**.

Manon-Jensen T, Kjeld NG , Karsdal MA (2016). Collagen-mediated hemostasis. *J Thromb Haemost*. **14**, 438-448.

Meester JA, Vandeweyer G, Pintelon I, Lammens M, Van Hoorick L, De Belder S, Waitzman K, Young L, Markham LW, Vogt J, Richer J, Beauchesne LM, Unger S, Superti-Furga A, Prsa M, Dhillon R, Reyniers E, Dietz HC, Wuyts W, Mortier G, Verstraeten A, Van Laer L , Loeys BL (2016). Loss-of-function mutations in the X-linked biglycan gene cause a severe syndromic form of thoracic aortic aneurysms and dissections. *Genet Med*.

Meienberg J, Rohrbach M, Neuenschwander S, Spanaus K, Giunta C, Alonso S, Arnold E, Henggeler C, Regenass S, Patrignani A, Azzarello-Burri S, Steiner B, Nygren AO, Carrel T, Steinmann B , Mátyás G (2010). Hemizygous deletion of COL3A1, COL5A2, and MSTN causes a complex phenotype with aortic dissection: a lesson for and from true haploinsufficiency. *Eur J Hum Genet*. **18**, 1315-1321.

Melrose J, Whitelock J, Xu Q , Ghosh P (1998). Pathogenesis of abdominal aortic aneurysms: possible role of differential production of proteoglycans by smooth muscle cells. *J Vasc Surg*. **28**, 676-686.

Meng YH, Tian C, Liu L, Wang L , Chang Q (2014). Elevated expression of connective tissue growth factor, osteopontin and increased collagen content in human ascending thoracic aortic aneurysms. *Vascular*. **22**, 20-27.

Micale L, Turturo MG, Fusco C, Augello B, Jurado LA, Izzi C, Digilio MC, Milani D, Lapi E, Zelante L , Merla G (2010). Identification and characterization of seven novel mutations of elastin gene in a cohort of patients affected by supravalvular aortic stenosis. *Eur J Hum Genet*. **18**, 317-323.

Munezane T, Hasegawa T, Suritala, Tanaka A, Okada K , Okita Y (2010). Activation of transglutaminase type 2 for aortic wall protection in a rat abdominal aortic aneurysm formation. *J Vasc Surg*. **52**, 967-974.

Nakajima N, Nagahiro S, Sano T, Satomi J , Satoh K (2000). Phenotypic modulation of smooth muscle cells in human cerebral aneurysmal walls. *Acta Neuropathol*. **100**, 475-480.

Neshige S, Takeshima S, Himeno T, Yoshimoto T, Takamatsu K, Morisaki Y , Kuriyama M (2014). [Marfan syndrome presenting with cervical tortuous arteries and subcortical hemorrhage: a case with a mutation in an intron of the FBN1 gene]. *Rinsho Shinkeigaku*. **54**, 897-902.

Nico B , Ribatti D (2012). Morphofunctional aspects of the blood-brain barrier. *Curr Drug Metab*. **13**, 50-60.

Niiyama T, Higuchi I, Hashiguchi T, Suehara M, Uchida Y, Horikiri T, Shiraishi T, Saitou A, Hu J, Nakagawa M, Arimura K , Osame M (2003). Capillary changes in skeletal muscle of patients with Ullrich's disease with collagen VI deficiency.ed^eds), pp. 137-142.

Osborne-Pellegrin MJ, Farjanel J , Hornebeck W (1990). Role of elastase and lysyl oxidase activity in spontaneous rupture of internal elastic lamina in rats. *Arteriosclerosis*. **10**, 1136-1146.

Paloschi V, Kurtovic S, Folkersen L, Gomez D, Wågsäter D, Roy J, Petrini J, Eriksson MJ, Caidahl K, Hamsten A, Liska J, Michel JB, Franco-Cereceda A , Eriksson P (2011). Impaired splicing of fibronectin is associated with thoracic aortic aneurysm formation in patients with bicuspid aortic valve. *Arterioscler Thromb Vasc Biol*. **31**, 691-697.

Papke CL , Yanagisawa H (2014). Fibulin-4 and fibulin-5 in elastogenesis and beyond: Insights from mouse and human studies. *Matrix Biol*. **37**, 142-149.

Perisic L, Aldi S, Sun Y, Folkersen L, Razuvaev A, Roy J, Lengquist M, Akesson S, Wheelock CE, Maegdefessel L, Gabrielsen A, Odeberg J, Hansson GK, Paulsson-Berne G , Hedin U (2016). Gene expression signatures, pathways and networks in carotid atherosclerosis. *J Intern Med*. **279**, 293-308.

Plaisier E, Chen Z, Gekeler F, Benhassine S, Dahan K, Marro B, Alamowitch S, Paques M , Ronco P (2010). Novel COL4A1 mutations associated with HANAC syndrome: a role for the triple helical CB3[IV] domain. *Am J Med Genet A*. **152A**, 2550-2555.

Ponticos M (2013). Connective tissue growth factor (CCN2) in blood vessels. *Vascul Pharmacol*. **58**, 189-193.

Ponticos M, Partridge T, Black CM, Abraham DJ , Bou-Gharios G (2004). Regulation of collagen type I in vascular smooth muscle cells by competition between Nkx2.5 and deltaEF1/ZEB1. *Mol Cell Biol*. **24**, 6151-6161.

Pradhan-Palikhe P, Vikatmaa P, Lajunen T, Palikhe A, Lepäntalo M, Tervahartiala T, Salo T, Saikku P, Leinonen M, Pussinen PJ , Sorsa T (2010). Elevated MMP-8 and decreased myeloperoxidase concentrations associate significantly with the risk for peripheral atherosclerosis disease and abdominal aortic aneurysm. *Scand J Immunol*. **72**, 150-157.

Ramirez F, Sakai LY, Rifkin DB , Dietz HC (2007). Extracellular microfibrils in development and disease. *Cell Mol Life Sci*. **64**, 2437-2446.

Rasi K, Piuhola J, Czabanka M, Sormunen R, Ilves M, Leskinen H, Rysä J, Kerkelä R, Janmey P, Heljasvaara R, Peuhkurinen K, Vuolteenaho O, Ruskoaho H, Vajkoczy P, Pihlajaniemi T , Eklund L (2010). Collagen XV is necessary for modeling of the extracellular matrix and its deficiency predisposes to cardiomyopathy. *Circ Res*. **107**, 1241-1252.

Remus EW, O'Donnell RE, Rafferty K, Weiss D, Joseph G, Csiszar K, Fong SF , Taylor WR (2012). The role of lysyl oxidase family members in the stabilization of abdominal aortic aneurysms. *Am J Physiol Heart Circ Physiol*. **303**, H1067-1075.

Ren P, Zhang L, Xu G, Palmero LC, Albini PT, Coselli JS, Shen YH , LeMaire SA (2013). ADAMTS-1 and ADAMTS-4 levels are elevated in thoracic aortic aneurysms and dissections. *Ann Thorac Surg*. **95**, 570-577.

Ruigrok YM, Rinkel GJ , Wijmenga C (2006). The versican gene and the risk of intracranial aneurysms. *Stroke*. **37**, 2372-2374.

Ruigrok YM, Rinkel GJ, Wijmenga C, Kasuya H, Tajima A, Takahashi T, Hata A, Inoue I , Krischek B (2009). Association analysis of genes involved in the maintenance of the integrity of the extracellular matrix with intracranial aneurysms in a Japanese cohort. *Cerebrovasc Dis*. **28**, 131-134.

Ruperez M, Lorenzo O, Blanco-Colio LM, Esteban V, Egido J , Ruiz-Ortega M (2003a). Connective tissue growth factor is a mediator of angiotensin II-induced fibrosis. *Circulation*. **108**, 1499-1505.

Ruperez M, Ruiz-Ortega M, Esteban V, Lorenzo O, Mezzano S, Plaza JJ , Egido J (2003b). Angiotensin II increases connective tissue growth factor in the kidney. *Am J Pathol*. **163**, 1937-1947.

Saracini C, Bolli P, Sticchi E, Pratesi G, Pulli R, Sofi F, Pratesi C, Gensini GF, Abbate R , Giusti B (2012). Polymorphisms of genes involved in extracellular matrix remodeling and abdominal aortic aneurysm. *J Vasc Surg*. **55**, 171-179.e172.

Sathyan S, Koshy LV, Balan S, Easwer HV, Premkumar S, Nair S, Bhattacharya RN, Alapatt JP , Banerjee M (2014). Association of Versican (VCAN) gene polymorphisms rs251124 and rs2287926 (G428D), with intracranial aneurysm. *Meta Gene*. **2**, 651-660.

Satoh K, Tsukamoto M, Shindoh M, Totsuka Y, Oda T , Matsumoto K (2010). Increased expression of tenascin-x in thoracic and abdominal aortic aneurysm tissues. *Biol Pharm Bull*. **33**, 1898-1902.

Schiffers PM, Henrion D, Boulanger CM, Colucci-Guyon E, Langa-Vuves F, van Essen H, Fazzi GE, Lévy BI , De Mey JG (2000). Altered flow-induced arterial remodeling in vimentin-deficient mice. *Arterioscler Thromb Vasc Biol*. **20**, 611-616.

Schildmeyer LA, Braun R, Taffet G, Debiasi M, Burns AE, Bradley A , Schwartz RJ (2000). Impaired vascular contractility and blood pressure homeostasis in the smooth muscle alpha-actin null mouse. *FASEB J*. **14**, 2213-2220.

Schulz B, Pruessmeyer J, Maretzky T, Ludwig A, Blobel CP, Saftig P , Reiss K (2008). ADAM10 regulates endothelial permeability and T-Cell transmigration by proteolysis of vascular endothelial cadherin. *Circ Res*. **102**, 1192-1201.

Song Y, Xie Y, Liu F, Zhao C, Yu R, Ban S, Ye Q, Wen J, Wan H, Li X, Ma R , Meng Z (2013). Expression of matrix metalloproteinase-12 in aortic dissection. *BMC Cardiovasc Disord*. **13**, 34.

Spin JM, Hsu M, Azuma J, Tedesco MM, Deng A, Dyer JS, Maegdefessel L, Dalman RL , Tsao PS (2011). Transcriptional profiling and network analysis of the murine angiotensin II-induced abdominal aortic aneurysm. *Physiol Genomics*. **43**, 993-1003.

Stegemann C, Didangelos A, Barallobre-Barreiro J, Langley SR, Mandal K, Jahangiri M , Mayr M (2013). Proteomic identification of matrix metalloproteinase substrates in the human vasculature. *Circ Cardiovasc Genet*. **6**, 106-117.

Sun C, Wu MH, Guo M, Day ML, Lee ES , Yuan SY (2010). ADAM15 regulates endothelial permeability and neutrophil migration via Src/ERK1/2 signalling. *Cardiovasc Res*. **87**, 348-355.

Takaluoma K, Hyry M, Lantto J, Sormunen R, Bank RA, Kivirikko KI, Myllyharju J , Soininen R (2007). Tissue-specific changes in the hydroxylysine content and cross-links of collagens and alterations in fibril morphology in lysyl hydroxylase 1 knock-out mice. *J Biol Chem*. **282**, 6588-6596.

Takayanagi T, Forrester SJ, Kawai T, Obama T, Tsuji T, Elliott KJ, Nuti E, Rossello A, Kwok HF, Scalia R, Rizzo V , Eguchi S (2016). Vascular ADAM17 as a Novel Therapeutic Target in Mediating Cardiovascular Hypertrophy and Perivascular Fibrosis Induced by Angiotensin II. *Hypertension*. **68**, 949-955.

Takeda N, Morita H, Fujita D, Inuzuka R, Taniguchi Y, Imai Y, Hirata Y , Komuro I (2015). Congenital contractural arachnodactyly complicated with aortic dilatation and dissection: Case report and review of literature. *Am J Med Genet A*. **167A**, 2382-2387.

Tang T, Thompson JC, Wilson PG, Yoder MH, Mueller J, Fischer JW, Williams KJ , Tannock LR (2014). Biglycan deficiency: increased aortic aneurysm formation and lack of atheroprotection. *J Mol Cell Cardiol*. **75**, 174-180.

Theocharis AD , Karamanos NK (2002). Decreased biglycan expression and differential decorin localization in human abdominal aortic aneurysms. *Atherosclerosis*. **165**, 221-230.

Thompson M , Cockerill G (2006). Matrix metalloproteinase-2: the forgotten enzyme in aneurysm pathogenesis. *Ann N Y Acad Sci*. **1085**, 170-174.

Toth P, Tarantini S, Springo Z, Tucsek Z, Gautam T, Giles CB, Wren JD, Koller A, Sonntag WE, Csiszar A , Ungvari Z (2015). Aging exacerbates hypertension-induced cerebral microhemorrhages in mice: role of resveratrol treatment in vasoprotection. *Aging Cell*. **14**, 400-408.

Ueda K, Yoshimura K, Yamashita O, Harada T, Morikage N , Hamano K (2015). Possible dual role of decorin in abdominal aortic aneurysm. *PLoS One*. **10**, e0120689.

Villano JS, Boehm CA, Carney EL , Cooper TK (2012). Complications of elastase-induced arterial saccular aneurysm in rabbits: case reports and literature review. *Comp Med*. **62**, 480-486.

Wagenseil JE , Mecham RP (2009). Vascular extracellular matrix and arterial mechanics. *Physiol Rev*. **89**, 957-989.

Walker LC, Overstreet MA, Siddiqui A, De Paepe A, Ceylaner G, Malfait F, Symoens S, Atsawasuwan P, Yamauchi M, Ceylaner S, Bank RA , Yeowell HN (2005). A novel mutation in the lysyl hydroxylase 1 gene causes decreased lysyl hydroxylase activity in an Ehlers-Danlos VIA patient. *J Invest Dermatol*. **124**, 914-918.

Wang M, Wang HH , Lakatta EG (2013). Milk fat globule epidermal growth factor VIII signaling in arterial wall remodeling. *Curr Vasc Pharmacol*. **11**, 768-776.

Wang X, LeMaire SA, Chen L, Shen YH, Gan Y, Bartsch H, Carter SA, Utama B, Ou H, Coselli JS , Wang XL (2006). Increased collagen deposition and elevated expression of connective tissue growth factor in human thoracic aortic dissection. *Circulation*. **114**, I200-205.

Whitlow CT, Geer CP, Mattern CW, Mussat-Whitlow BJ, Yazdani SK, Berry JL, Lalli JH, Claus RO, Challa VR , Morris PP (2009). Endovascular histologic effects of ultrathin gold- or vitronectin-coated platinum aneurysm coils in a rodent arterial occlusion model: a preliminary investigation. *AJNR Am J Neuroradiol*. **30**, 85-90.

Wilson WR, Schwalbe EC, Jones JL, Bell PR , Thompson MM (2005). Matrix metalloproteinase 8 (neutrophil collagenase) in the pathogenesis of abdominal aortic aneurysm. *Br J Surg*. **92**, 828-833.

Wint DP, Butman JA, Masdeu JC, Meyer-Lindenberg A, Mervis CB, Sarpal D, Morris CA , Berman KF (2014). Intracranial arteries in individuals with the elastin gene hemideletion of Williams syndrome. *AJNR Am J Neuroradiol*. **35**, 90-94.

Yanagisawa H, Davis EC, Starcher BC, Ouchi T, Yanagisawa M, Richardson JA , Olson EN (2002). Fibulin-5 is an elastin-binding protein essential for elastic fibre development in vivo. *Nature*. **415**, 168-171.

Yeowell HN , Walker LC (2000). Mutations in the lysyl hydroxylase 1 gene that result in enzyme deficiency and the clinical phenotype of Ehlers-Danlos syndrome type VI. *Mol Genet Metab*. **71**, 212-224.

Yuan SM, Wang J, Huang HR , Jing H (2011). Osteopontin expression and its possible functions in the aortic disorders and coronary artery disease. *Rev Bras Cir Cardiovasc*. **26**, 173-182.

Zanetti M, Braghetta P, Sabatelli P, Mura I, Doliana R, Colombatti A, Volpin D, Bonaldo P , Bressan GM (2004). EMILIN-1 deficiency induces elastogenesis and vascular cell defects. *Mol Cell Biol*. **24**, 638-650.

Zhan B, Hu Z, Chen J, Zhu R, Zhao H, Yang J, Zhang Z , Nie R (2015). KLF15 Overexpression Protects beta-Aminopropionitrile-Induced Aortic Rupture in Rodent Model via Inhibiting Connective Tissue Growth Factor. *Thorac Cardiovasc Surg*.

Zhang K, Pan X, Zheng J, Xu D, Zhang J , Sun L (2015). Comparative tissue proteomics analysis of thoracic aortic dissection with hypertension using the iTRAQ technique. *Eur J Cardiothorac Surg*. **47**, 431-438.

Zhang T, Xu J, Li D, Chen J, Shen X, Xu F, Teng F, Deng Y, Ma H, Zhang L, Zhang G, Zhang Z, Wu W, Liu X, Yang M, Jiang B , Guo D (2014). Salvianolic acid A, a matrix metalloproteinase-9 inhibitor of Salvia miltiorrhiza, attenuates aortic aneurysm formation in apolipoprotein E-deficient mice. *Phytomedicine*. **21**, 1137-1145.

Zhu H, Cao M, Figueroa JA, Cobos E, Uretsky BF, Chiriva-Internati M , Hermonat PL (2014). AAV2/8-hSMAD3 gene delivery attenuates aortic atherogenesis, enhances Th2 response without fibrosis, in LDLR-KO mice on high cholesterol diet. *J Transl Med*. **12**, 252.

Ziganshin BA, Bailey AE, Coons C, Dykas D, Charilaou P, Tanriverdi LH, Liu L, Tranquilli M, Bale AE , Elefteriades JA (2015). Routine Genetic Testing for Thoracic Aortic Aneurysm and Dissection in a Clinical Setting. *Ann Thorac Surg*. **100**, 1604-1611.

Zinnanti WJ, Lazovic J, Housman C, Antonetti DA, Koeller DM, Connor JR , Steinman L (2014). Mechanism of metabolic stroke and spontaneous cerebral hemorrhage in glutaric aciduria type I. *Acta Neuropathol Commun*. **2**, 13.
